# Ionic Double‐Network Hydrogels for Integrated Electromagnetic Shielding and Self‐Powered Sensing in Wearable Electronics

**DOI:** 10.1002/advs.202509115

**Published:** 2025-06-30

**Authors:** Chenchen Wang, Yao Ding, Tianzhao Wu, Zihua Li, Chuanshuang Hu, Zhuoqun Wang, Yonghui Zhou, Xiuyi Lin, Weiwei Zhang, Jiangtao Xu

**Affiliations:** ^1^ Key Laboratory of Advanced Materials for Facility Agriculture Ministry of Agriculture and Rural Affairs College of Materials and Energy South China Agricultural University No. 483 Wushan Road Guangzhou 510642 China; ^2^ State Key Laboratory of Advanced Forming Technology and Equipment China Academy of Machinery Science and Technology No. 2 Shouti South Road Beijing 100000 China; ^3^ Academy of Interdisciplinary Studies on Intelligent Molecules Tianjin Key Laboratory of Structure and Performance for Functional Molecules College of Chemistry Tianjin Normal University Tianjin 300387 China

**Keywords:** absorption dominance, electromagnetic interference shielding, ionic conductive hydrogel, sensor monitoring

## Abstract

Cardiovascular implantable electronic devices (CIEDs) face dual challenges of high‐frequency electromagnetic interference and functional integration. This work reports a multifunctional material constructed via a double‐network ionic hydrogel strategy, enabling the integrated realization of efficient electromagnetic shielding and self‐powered physiological monitoring. An interpenetrating network skeleton is formed through physical crosslinking of sodium alginate (SA) with Ca^2^⁺ and in situ polymerization of acrylamide (AM). By regulating the specific coordination of ions to induce directional channels and synergistically regulating salt concentration with hydration, an absorption‐dominated shielding mechanism centered on ion polarization‐interface relaxation is established. The optimized *h*‐CA‐PAM‐Li⁺‐1.0 hydrogel exhibits an electromagnetic interference (EMI) shielding effectiveness (SE_T_) of 63.75 dB in the X‐band, with absorption loss accounting for over 93%. Leveraging the excellent ionic conductivity of the hydrogel, a self‐powered sensor encapsulated in PDMS films and integrated with wireless modules is fabricated, capable of real‐time capture of physiological signals such as heartbeat while maintaining high sensitivity and anti‐interference capability in dynamic environments. Free of traditional conductive fillers, this material combines biocompatibility, low cost, and designability, providing a material‐device‐system integrated solution for electromagnetic protection and intelligent monitoring of implantable electronic devices and opening a new research paradigm for multifunctional shielding materials.

## Graphical abstract

A multifunctional dual network ionic hydrogel is used to address the challenges of EMI and health detection integration for CIEDs. An absorption‐based EMI shielding mechanism is achieved through ion polarization/interfacial relaxation. With an optimal SE of 63.75 dB and absorption loss greater than 93%, the wirelessly integrated self‐powered sensor in a PDMS package captures physiological signals.

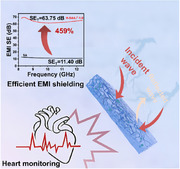

## 1 Introduction

Cardiovascular implantable electronic devices (CIEDs), such as cardiac pacemakers, have saved the lives of millions of patients worldwide as core implantable devices for cardiac treatment.^[^ ^1^, ^2^ ^]^ However, the high‐frequency electromagnetic environment in modern society—including 5G communications and electromagnetic radiation from medical equipment—poses a severe threat to the normal operation of these implanted devices.^[^ ^3^ ^]^ Studies indicate that 25% of pacemaker malfunctions are directly associated with external electromagnetic interference (EMI).^[^ ^3^ ^]^ Although traditional electromagnetic shielding solutions (e.g., metal coatings, conductive polymers) can suppress interference, these approaches are characterized by two intrinsic limitations. The primary challenge in biocompatibility arises from metal‐based shielding materials, whose rigid interfaces are prone to eliciting tissue inflammatory responses and whose prolonged in vivo implantation may induce galvanic corrosion, as demonstrated by preclinical assessments of metallic implant coatings.^[^ ^4^ ^]^ A concurrent limitation is functional specialization, whereby existing materials solely provide electromagnetic shielding while lacking the capability for real‐time monitoring of cardiac physiological signals, a shortfall that directly contradicts the escalating clinical requirement for implanted devices integrating protective and diagnostic functionalities, as emphasized in recent reviews of advanced medical devices.^[^ ^5^ ^]^ To overcome these limitations, a non‐invasive skin‐adhesive alternative is used that eliminates the risk of implantation while maintaining EMI shielding. This wearable design avoids long‐term biocompatibility issues and enables comfortable cardiovascular monitoring, and is particularly suitable for patients who require temporary or continuous assessment without invasive procedures.

Recently, hydrogels have attracted increasing attention and have been applied in many cutting‐edge technological fields, due to excellent flexibility, ionic conductivity, and biocompatibility. Water, as a highly polar liquid, has been widely demonstrated for its high static dielectric constant and strong polarization loss.^[^ ^6^ ^]^ It can be inferred that a superior attenuation capability to EMW could be achieved by the swollen hydrogel.^[^ ^7^, ^8^ ^]^ However, it is difficult for pure hydrogels to meet the EMI shielding requirements. Therefore, it is necessary to develop EMI shielding materials with a high conductivity of hydrogels. Currently, various types of conductive hydrogels have been developed based on different conducting mechanisms, such as ions, carbon nanomaterials, metal nanomaterials, conducting polymers, etc.^[^ ^9^, ^10^, ^11^ ^]^ Zhou et al.^[^ ^10^ ^]^ prepared PEDOT:PSS hydrogels using PEDOT and PSS as conductive fillers. The excellent conductivity of the hydrogel resulted in superior EMI shielding performance, with an EMI shielding effectiveness (SE) of 81.2 dB. Subsequently, Zhu et al.^[^ ^12^ ^]^ introduced MXene into PEDOT:PSS hydrogels, thereby enriching the conductive system (electronic and ionic conduction) of the hydrogels. Notably, the EMI SE attained a level of 99.99%, even when the filler content was as low as 1.8 wt.%. In addition, Diao et al.^[^ ^13^ ^]^ doped CNTs into polyacrylamide (PAM)/polyethylene glycol (PEG) hydrogel. The EMI SE of the CNTs/PAM/PEG conductive hydrogel was up to 32.92 dB. Fang et al.^[^ ^11^ ^]^ combined PVA, MXene, and glycerol into an organic conductive hydrogel. Its EMI SE reached 42.34 dB in the X‐band, exhibiting 99.9% absorption rate. Besides, highly conductive 1‐ethyl‐3‐methylimidazole ethyl sulfate ([EMI][ES]) ionic liquids have been used to prepare EMI shielding materials.^[^ ^14^ ^]^ Despite these advances, a key challenge remains: current hydrogel‐based materials often require expensive and difficult‐to‐process conductive fillers to achieve both high conductivity and effective EMI shielding. This approach inevitably compromises intrinsic hydrogel advantages such as superior compliance, biocompatibility, and deformability. Critically, while ionically conductive hydrogels without fillers represent an elegant solution to preserve biocompatibility and mechanical properties, recent reports in this domain often demonstrate limited EMI SE (48.32 dB) or fail to elucidate and leverage unique mechanisms beyond basic ionic conductivity.^[^ ^8^, ^12^ ^]^

In addition, although there are numerous reports on hydrogel‐based sensors and their excellent properties^[^ ^15^ ^]^ a significant disconnect exists between EMI shielding and sensing functionalities in current hydrogel materials. These functions typically exist in separate components or devices, lacking a cohesive, integrated design. This separation results in bulky, complex structures, increased assembly and maintenance costs, and crucially, prevents the simultaneous delivery of electromagnetic protection and physiological monitoring in a single, streamlined platform. This functional segregation severely limits the practical utility and application scope of devices aiming for integrated protection and monitoring.

To tackle the existing challenges in integrating EMI shielding with sensing functionalities, this study proposes a design strategy of double‐network ionic hydrogel to construct a multifunctional material combining high‐efficiency EMI shielding and self‐powered sensing. This strategy achieves performance breakthroughs through three aspects, including dual‐network structure construction, ion coordination regulation, and functional integration design. Replacing conventional conductive fillers, cost‐effective ionic salt solutions are employed as functional carriers. By synergistically modulating the salt concentration and hydration effect, an efficient electromagnetic absorption mechanism dominated by strong ion polarization‐interfacial relaxation is constructed and enhanced. Lithium ion (Li⁺) doping combined with hydration treatment optimizes the ionic concentration gradient within the hydrogel, simultaneously enhancing electrical conductivity and dielectric loss. The specific coordination between G‐block units in calcium alginate chains and Li⁺ enables directional ion transport, creating ordered ionic channels. The double‐network structure further optimizes ion migration pathways, while hydration‐induced ion distribution heterogeneity strengthens interfacial relaxation effects, thereby achieving efficient electromagnetic wave absorption and energy dissipation, and laying the foundation for electrical signal transmission. Finally, leveraging the hydrogel's excellent ionic conductivity, self‐powered sensing electrodes are fabricated by encapsulating the hydrogel within polydimethylsiloxane (PDMS) films and integrating wireless transmission modules, resulting in an integrated protective monitoring device suitable for cardiovascular implantable electronic equipment. The device not only shields electromagnetic interference to ensure normal device operation but also enables real‐time physiological signal monitoring, providing a novel paradigm for developing low‐cost and sustainable multifunctional shielding materials. This provides a novel, non‐implantable, integrated solution for the protection and monitoring of cardiovascular implantable electronic devices.

## 2 Results and Discussion

### 2.1 Preparation and Characterization of Double Network Ionic Hydrogel

**Figure 1:**
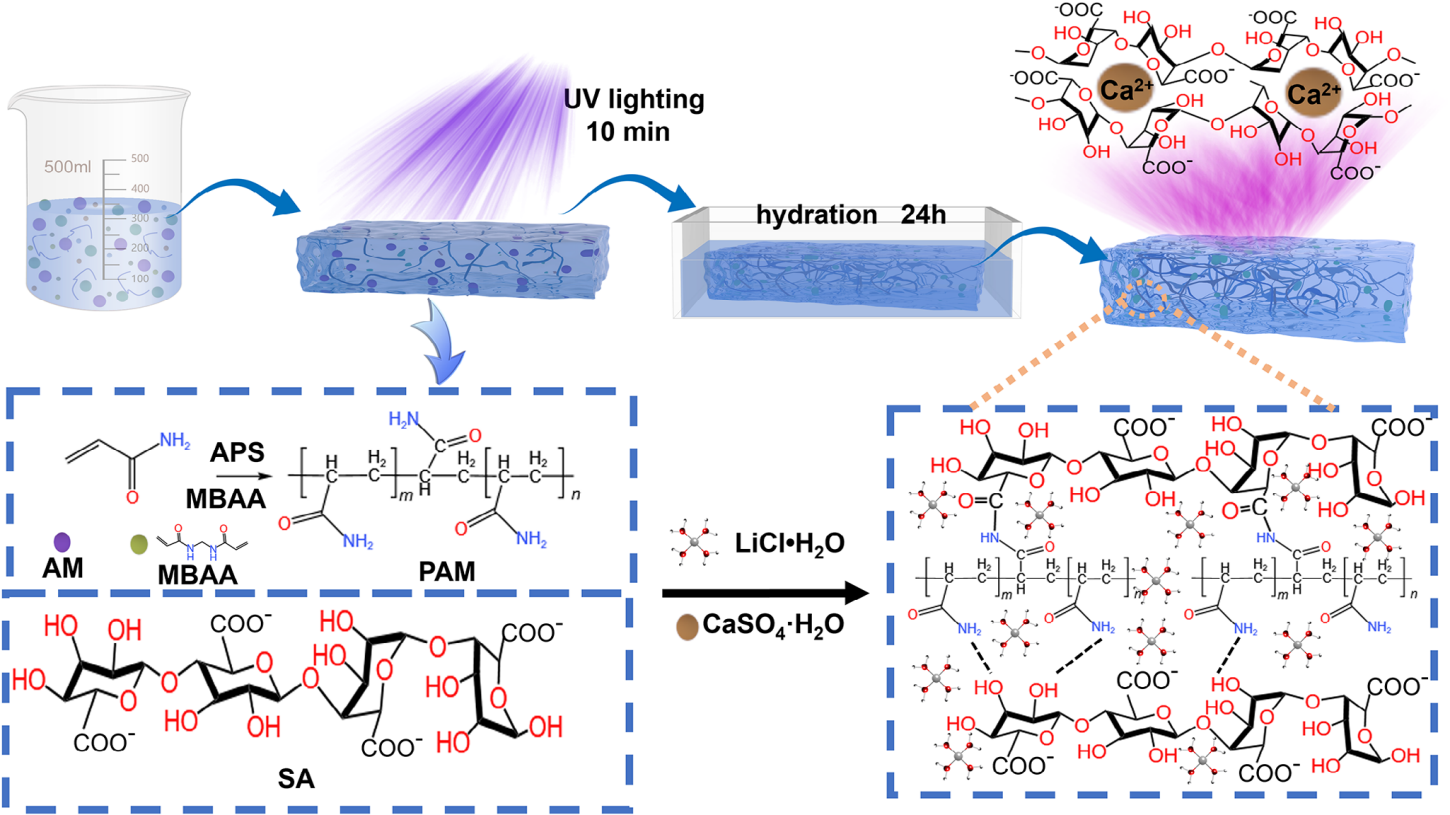
Schematic and design of the CA‐PAM‐Li^+^ and *h*‐CA‐PAM‐Li^+^ hydrogels.

Double network ionic hydrogels of CA‐PAM‐Li^+^ and *h*‐CA‐PAM‐Li^+^ doped with different Li^+^ (0.5, 1.0, 1.5, and 2.0 m) were prepared by in situ coordination and polymerization (**Figure** **1**). In the designed hydrogel system, the AM monomer undergoes free radical polymerization to form a PAM first network that is covalently cross‐linked. Meanwhile, the Ca^2+^ in the system and the carboxyl groups on the sodium alginate chain are coordinated to form a second network of ionic cross‐linking. Besides, the amino group of PAM and the hydroxyl group of calcium alginate (CA) were utilized to form hydrogen bonds, thereby strengthening the intermolecular interactions. This resulted in the linear CA molecules being interspersed in the PAM network, thereby forming a double network structure. The specific coordination between G‐block units in CA chains and Li⁺ enables directional ion transport, creating ordered ionic channels, and the double‐network structure further optimizes ion migration pathways, thus improving the conductivity of the hydrogel. The prepared hydrogels (CA‐PAM‐Li^+^) were subsequently hydrated in aqueous solutions with different ionic concentrations, forming the *h*‐CA‐PAM‐Li^+^ hydrogels.

The FTIR was utilized to characterize the structural and compositional properties of the *h*‐CA‐PAM‐Li^+^ hydrogels (Figure S1, Supporting Information). A broad peak at 3310 cm^−1^ is clearly observed, indicating the presence of bound hydroxyl groups and NH_2_ stretching vibrations in the sample.^[^ ^16^ ^]^ The peak ≈1436 cm^−1^ is attributed to the bending vibration of methylene (─CH_2_─) produced by the polymerization reaction of acrylamide monomers.^[^ ^17^ ^]^ The peak of the asymmetric stretching vibration at 1623cm^−1^ is attributed to the carboxyl group of sodium alginate, which is defined as the ─COO─ group.^[^ ^18^ ^]^ The absorption peak at 1029 cm^−1^ is attributed to the C─O─C stretching vibration of alginate. The characterization results of FTIR spectroscopy indicate the successful preparation of CA‐PAM hydrogel. In addition, the absorption at 3310 cm^−1^ of the CA‐PAM‐Li^+^‐1.0 sample was significantly enhanced after hydration (Figure S1b, Supporting Information), indicating that more hydrogen bonds were formed after the introduction of water molecules, which resulted in the broadening of the ─OH peak and an increase in its intensity. The enhancement of the ─OH peak after hydration confirms that the water molecules successfully entered the material system and formed a hydrogen bonding network with polar groups (amide, carboxylic acid).^[^ ^19^ ^]^ Meanwhile, the peaks of the hydrogel in the low wavenumber region of 500–800 cm^−1^ disappeared after hydration. This suggests that hydration may affect its coordination structure so that Li^+^ binds more to water molecules to improve the mobility of ions, which is also reflected in the enhancement of the conductivity of the hydrogel after hydration.

**Figure 2:**
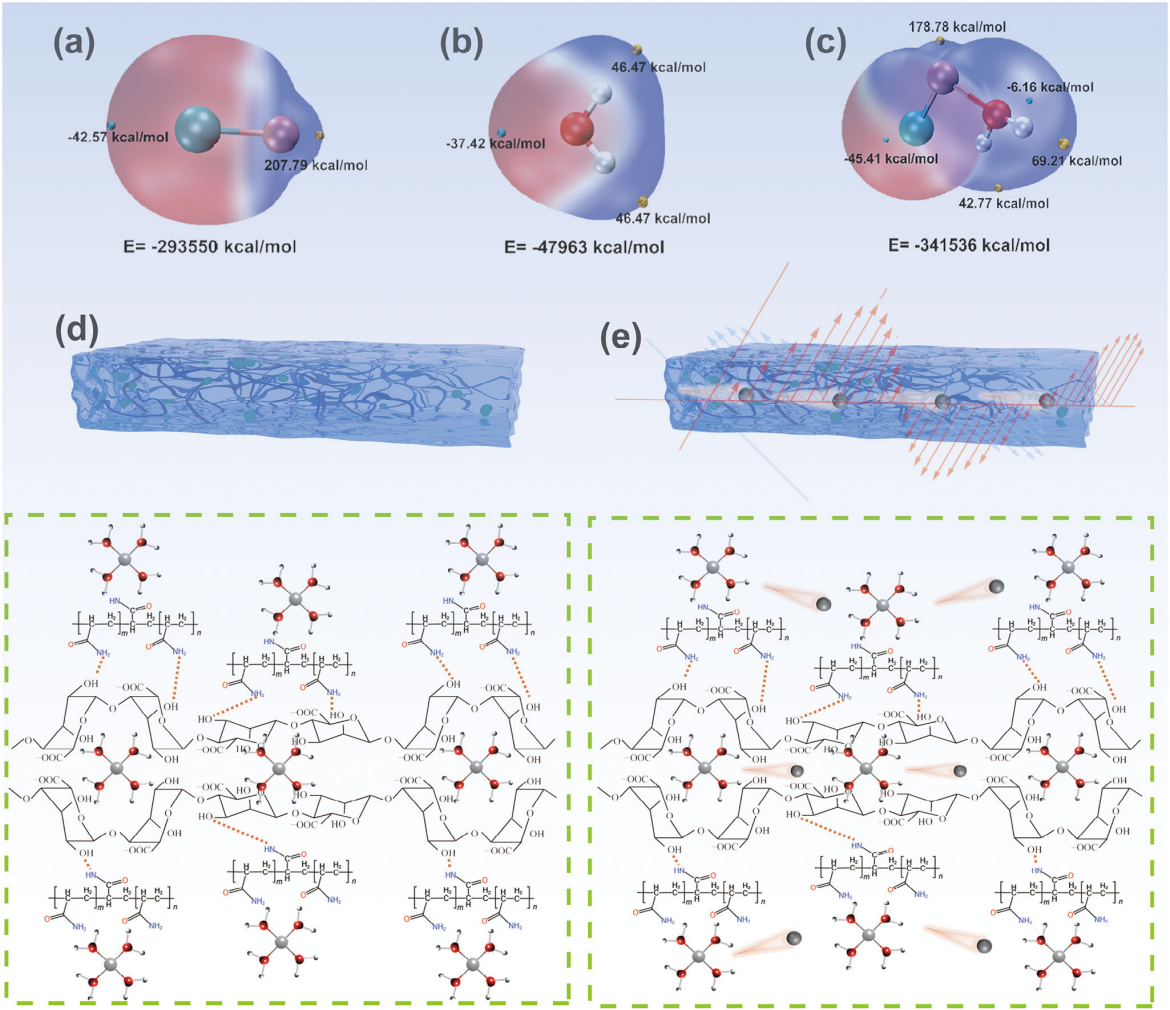
The surface electrostatic potential of a) LiCl, b) H_2_O, and c) LiCl‐H_2_O, d) and e) Ionic conductive pathways in ionic hydrogel systems.

In contrast to the conventional conductive filler‐based percolation mechanism, the ionic conduction in this system predominantly arises from the 3D transport network constructed by hydrocarbon molecular chains and their interchain spaces. The distance between Cl⁻ and H atoms in neighboring water molecules within the LiCl‐H₂O structure is 0.253 nm, whereas the distance between Li⁺ and O atoms in adjacent water molecules is 0.189 nm (Figure S2, Supporting Information). This shorter Li⁺‐O distance indicates stronger interactions between Li⁺ and water molecules, allowing more efficient charge transfer from Li⁺ to the water molecules. The composite network formed by cross‐linking CA and PAM molecules contains abundant hydrophilic groups (─OH, ─COOH, ─NH₂) on its surface (**Figure** **2**). These polar groups immobilize water molecules through hydrogen bonding, establishing a continuous 3D free‐volume network within the gel that provides coherent multiscale ion migration channels.

Notably, the G‐block units in calcium alginate molecular chains exhibit specific coordination with Li⁺.^[^ ^20^ ^]^ The formed coordination bonds not only facilitate directional ion transport but also create ordered molecular‐scale ion channels. Although some carboxyl groups bind to Ca^2^⁺, the uncoordinated carboxyl groups effectively trap Li⁺. This dual coordination mechanism promotes the formation of oriented ion transport paths between G‐blocks under an electric field (Figure 2e). Additionally, the amino groups in PAM chains bind water molecules through hydrogen‐bonding networks, generating localized microenvironments with high concentrations of hydrated Li⁺. Such localized hydration reduces the Li⁺ desolvation energy barrier, enabling electric‐field‐driven Li⁺ migration along PAM chains.^[^ ^21^ ^]^ The swelling behavior of the gel network further modulates conductivity. Hydration increases molecular chain spacing, enlarging free volume for ion migration and allowing more carriers to participate synchronously in conduction under an electric field. Consequently, the conductivity of *h*‐CA‐PAM‐Li^+^ hydrogels was enhanced from 11.85–43.12 to 19.14–51.48 S cm^−1^, compared to the CA‐PAM‐Li^+^ hydrogels. Moreover, hydration treatment enhances the complexity and heterogeneity of electrostatic potential distribution on LiCl‐H₂O surfaces (Figure 2c). This nonuniform charge distribution favors electromagnetic wave attenuation through dipole polarization losses.^[^ ^22^ ^]^

### 2.2 EMI Shielding Performance of CA‐PAM‐Li^+^ Hydrogels with Different Li^+^ Concentration

**Figure 3:**
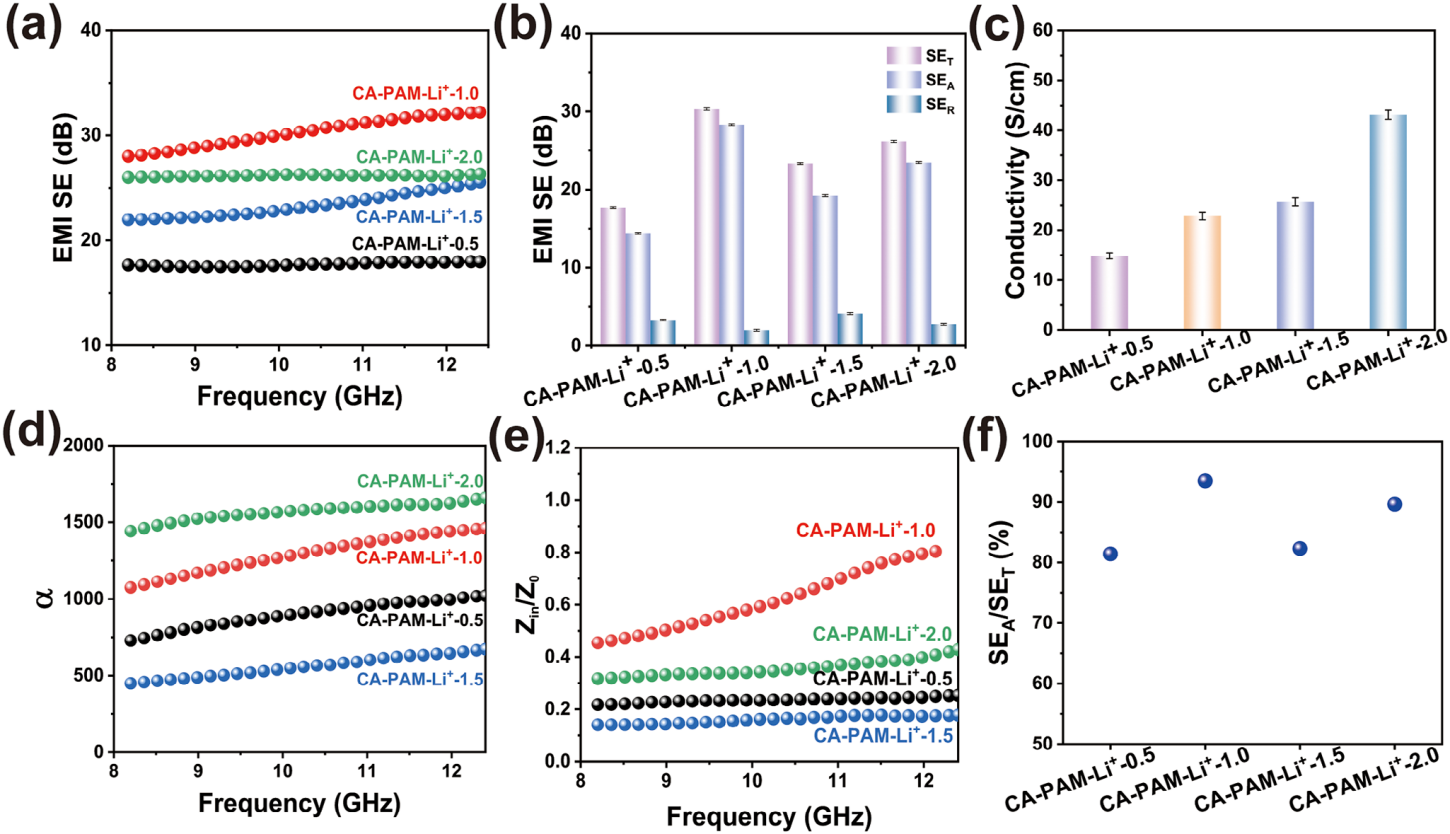
a,b) EMI shielding performance of CA‐PAM‐Li^+^ hydrogels with different Li^+^ concentrations in X‐band. c) Conductivity. d) Attenuation constant. e) Impedance matching. f) SE_A_/SE_T_.

Notwithstanding the fact that water possesses high dielectric properties, which have been demonstrated to result in attenuation of EMWs.^[^ ^6^ ^]^ However, the single loss mechanism that is present in water limits its ability to attenuate EMWs to a considerable extent.^[^ ^23^ ^]^ Consequently, the average SE_T_ of pure CA‐PAM hydrogel is a mere 11.40 dB, which is significantly below the standard (≥20 dB) for commercial utilization (Figure S3, Supporting Information). **Figure** **3**a demonstrates the EMI shielding performance of CA‐PAM‐Li^+^ hydrogels in the X‐band with varying LiCl concentrations. Incorporating LiCl has been demonstrated to enhance the EMI shielding performance of hydrogels. Among the hydrogels, the CA‐PAM‐Li^+^‐1.0 hydrogel, containing 1.0 m LiCl, exhibited the most effective EMI shielding, with SE_T_ of up to 30.29 dB. This is a 165.70% increase compared to the shielding performance of the CA‐PAM hydrogel. The primary cause of this phenomenon lies in the high conductivity of LiCl within the hydrogels. This high conductivity notably strengthens the interactions with EMWs, thereby resulting in increased EMW attenuation.^[^ ^8^ ^]^ As for the shielding materials, conductivity is a critical factor that influences EMI shielding effectiveness.^[^ ^23^ ^]^ The conductivity of CA‐PAM‐Li^+^ hydrogels is demonstrated in Figure S4 (Supporting Information) and Figure 3c. The conductivity of the CA‐PAM‐Li^+^ hydrogels exhibited an increase from 14.85 to 43.12 S cm^−1^ with the increase of LiCl concentration. The increasing LiCl concentration leads to a higher carrier density in the solution,^[^ ^24^ ^]^ which improves the conductivity by 2.9 times.

Interestingly, the EMI shielding performance of CA‐PAM‐Li^+^ hydrogels did not improve with the increase in conductivity. This prompts us to further explore the intrinsic reasons. Qualitative analysis of EMW attenuation properties of ionic hydrogels using attenuation constant (α) and impedance matching (Z). The α is commonly used to evaluate the electromagnetic loss characteristics of a material and is calculated as follows:^[^ ^25^ ^]^

(1)

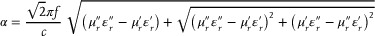




The *α*‐curve of the CA‐PAM‐Li^+^ hydrogels is depicted in Figure 3d. It has been demonstrated that CA‐PAM‐Li^+^‐2.0 exhibits optimal conductivity, resulting in the most substantial loss constant. However, the α (>1000) is obtained for CA‐PAM‐Li^+^‐1.0, despite its conductivity being smaller than that of CA‐PAM‐Li^+^‐1.5. It can be hypothesized that the increased entry of EMWs into the interior of SA/Li^+^‐1.0 for loss is the reason for the rise in its α value.

Impedance matching is typically employed to characterize the probability of EMWs penetrating the interior of the material. Good impedance matching allows more EMWs to penetrate inside the absorber, thus reducing surface EMWs reflections. Impedance matching can be calculated using the following formulation:^[^ ^26^ ^]^

(2) $$Z=\left|\frac{Z_{in}}{Z_{0}}\right|=\sqrt{\frac{\mu_{r}}{\varepsilon_{r}}}\;tanh\left[j\left(\frac{2\pi }{c}\right)fd\sqrt{\varepsilon_{r}\mu_{r}}\right]$$

where *d* is the thickness of the absorber, and *Z*_0_ and *Z*_in_ are the free space and input impedances of the absorber. When *Z* approaches 1 (in the range of 0.8–1.2), more EMWs are absorbed rather than reflected on the surface, which is considered a good impedance matching.^[^ ^26^ ^]^ The impedance matching for the samples are presented in Figure 3e. The optimal impedance matching performance of the CA‐PAM‐Li⁺‐1.0 hydrogel lies between 0.4 and 0.9, due to its proper electrical conductivity achieved by doping with lithium chloride (Figure 3c). The propriety electrical conductivity strengthens the impedance matching performance of the material and effectively avoids excessive reflection and transmission effects of EMWs, thus facilitating the dissipation of EMWs into the interior of the material. Consequently, the harmonization of the conductive and impedance matching capabilities culminated in an optimal EMI shielding outcome for CA‐PAM‐Li^+^‐1.0 hydrogel.

In addition, constructing micro‐pores in shielding architectures can significantly improve EMI shielding effectiveness by promoting multiple reflections of incident EMW.^[^ ^27^ ^]^ The SEM was used to characterize the microstructure of hydrogels doped with varying concentrations of LiCl (Figure S5, Supporting Information). As the concentration of LiCl in the hydrogel increased from 0.5 to 2.0 m, there was a gradual increase in the number of ionized beams inside the hydrogel (Figure S5e–h, Supporting Information). The increase in ionized beams means that there will be an increase in the conductivity of the hydrogel. Moreover, the microscopic morphology of the hydrogel transitions from a fine‐fragmented laminar structure (CA‐PAM‐Li^+^‐0.5) to a honeycomb structure (CA‐PAM‐Li^+^‐1.0) and ultimately to a less porous gel structure (CA‐PAM‐Li^+^‐1.5 and CA‐PAM‐Li^+^‐2.0). The change in the microstructure is attributed to the fact that introducing LiCl raises the proportion of hydrogen bonds in the water molecules and enhances the interaction forces between water molecules and/or hydrated ions.^[^ ^7^ ^]^ Consequently, an increase in LiCl concentration enhances the aggregation of molecular chains through a salting‐out effect or physical entanglement, thereby reducing the microporous structure of the hydrogel. The honeycomb structure of CA‐PAM‐Li^+^‐1.0 hydrogels increases the propagation path of EMWs, resulting in increased multiple reflection and scattering properties.^[^ ^28^ ^]^

The unique microporous structure and impedance‐matching properties of CA‐PAM‐Li^+^‐1.0 hydrogel contribute significantly to its SE_A_, which accounts for up to 93.49% of the SE_T_ (Figure 3f). To further confirm that the CA‐PAM‐Li^+^‐1.0 hydrogel can reduce the secondary reflection of EMWs, its reflection coefficient (R) and absorption coefficient (A) were calculated and analyzed. Figure S6 (Supporting Information) presents the power coefficient of CA‐PAM‐Li^+^ hydrogel. CA‐PAM‐Li^+^‐1.0 hydrogel has the largest A‐value (0.63), which is mainly due to its optimal impedance‐matching properties. When EMWs are incident on the surface of the material, more EMWs are able to enter the interior of the material for absorption loss, thus increasing its A‐value. In addition, the A‐value of CA‐PAM‐Li^+^‐1.0 hydrogel is significantly larger than the R‐value (0.3), which indicates that CA‐PAM‐Li+‐1.0 hydrogel is an absorption‐dominated EMI shielding mechanism. Absorption is the main EMI shielding material, which can effectively reduce the secondary pollution caused by incident EMWs due to reflection from the surrounding environment.

### 2.3 Effect of Hydration on the EMI Shielding Performance of *h*‐CA‐PAM‐Li^+^ Hydrogels

**Figure 4:**
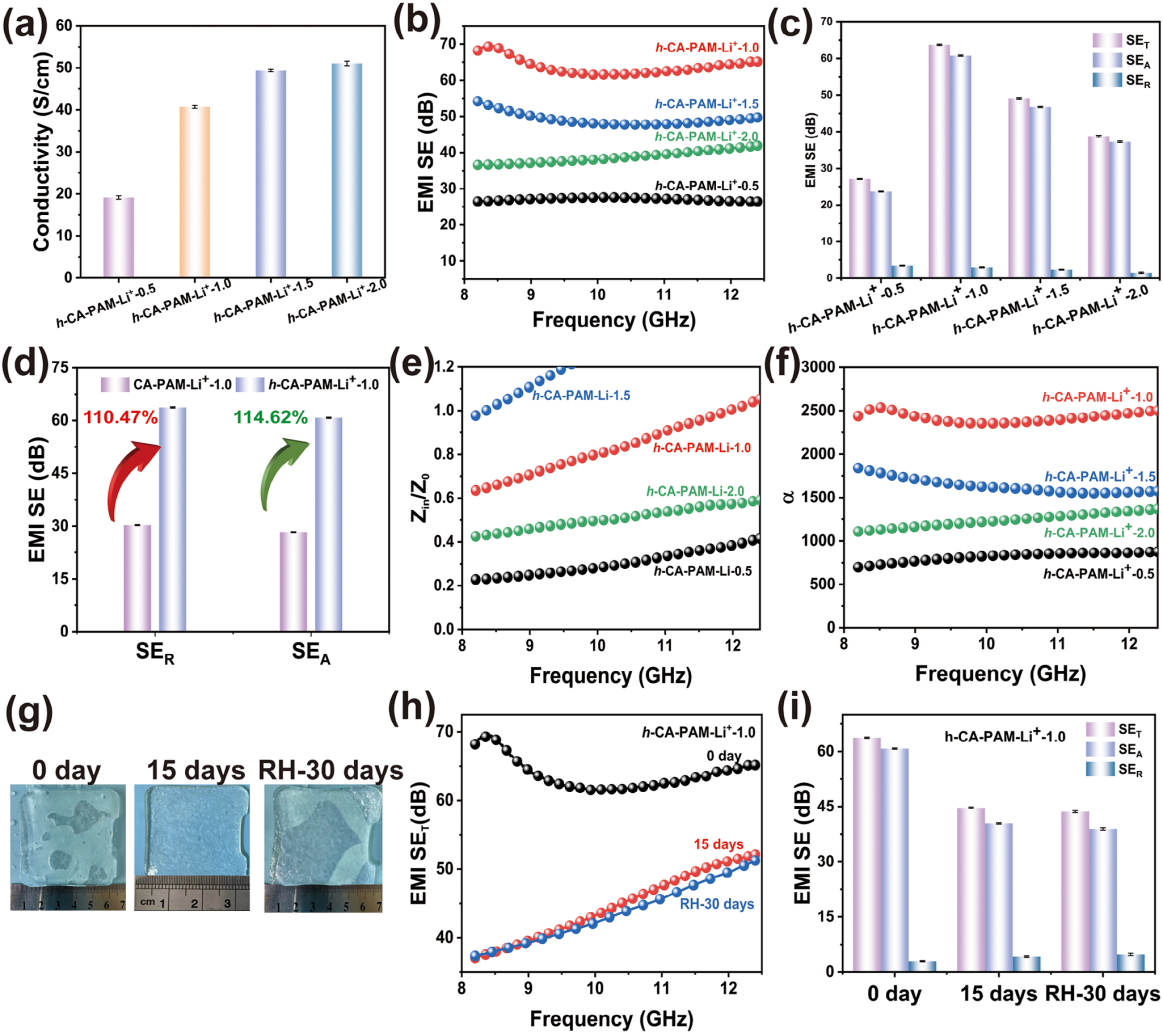
a) Conductivity. b,c) EMI shielding performance of *h*‐CA‐PAM‐Li^+^ hydrogels with different Li^+^ concentration in X‐band. d) Comparison of *h*‐CA‐PAM‐Li^+^‐1.0 and CA‐PAM‐Li^+^‐1.0 hydrogels' properties. e) Impedance matching. f) Attenuation constant. g) Physical picture of hydrogels left for different times. h,i) EMI shielding performance of *h*‐CA‐PAM‐Li^+^ hydrogels left for different times.

The addition of salt ions to hydrogels results in the formation of aggregated hydrogel networks that may inhibit ionic conductivity.^[^ ^9^ ^]^ Consequently, a strategy of hydrating the hydrogel in a salt solution of the corresponding concentration was adopted to further swell the cross‐linked network, so as to further enhance its electrical conductivity. The conductivity of *h*‐CA‐PAM‐Li^+^ hydrogels exhibited a similar trend to that of CA‐PAM‐Li^+^ hydrogels, thereby substantiating the pivotal function of Li^+^ in enhancing the conductivity of hydrogels, as demonstrated in **Figures** **4**a and S7 (Supporting Information). The primary reason for this phenomenon is the observation that the hydrogel undergoes swelling during the hydration process in the salt solution. This swelling process weakens the interactions between the polymerizations and improves the migration rate of the ions.^[^ ^29^ ^]^

The EMI shielding performance of *h*‐CA‐PAM‐Li^+^ hydrogel was further characterized after investigating its conductivity, as shown in Figure 4b,c. Following the hydration treatment, the EMI shielding performance of *h*‐CA‐PAM‐Li^+^ hydrogels was significantly improved. The average SE_T_ for *h*‐CA‐PAM‐Li^+^‐0.5, *h*‐CA‐PAM‐Li^+^‐1.0, *h*‐CA‐PAM‐Li^+^‐1.5, and *h*‐CA‐PAM‐Li^+^‐2.0 were 27.10, 63.75, 49.13, and 38.78 dB, respectively (Figure 4c). The SE_R_ were 3.44, 2.97, 2.38, and 1.52 dB, respectively. This indicates that its efficient EMI shielding effectiveness comes mainly from the absorption loss. The SE_T_ and SE_A_ of *h*‐CA‐PAM‐Li^+^‐1.0 hydrogels were increased by 110.4% and 114.62%, compared to CA‐PAM‐Li^+^‐1.0 hydrogel (Figure 4d). This is attributed to more EMWs being lost inside the hydrogel. The impedance matching of the *h*‐CA‐PAM‐Li^+^‐1.0 hydrogel was within the range from 0.6 to 1.0, corresponding to the distribution of the perfect matching interval (0.8–1.2).^[^ ^30^ ^]^ At this point, the EMWs incident on the surface of the hydrogel can be transmitted into its interior. Following hydration, the hydrogel exhibits enhanced ionic conductivity, thereby augmenting the conduction loss.^[^ ^11^ ^]^ Furthermore, LiCl has been demonstrated to enhance the strength of the interaction between the EMWs of the material, thereby attenuating the EMWs.^[^ ^8^ ^]^ The beneficial effect of LiCl in GHz wave interaction is further proved by the attenuation coefficient (Figure 4f). The *h*‐CA‐PAM‐Li^+^‐1.0 hydrogel has exhibited superior attenuation loss performance, a consequence of its effective conductive loss and the depolarization facilitated by a greater number of water molecules.^[^ ^7^ ^]^

To further illustrate the important role that water plays in hydrogels, the EMI shielding performance of hydrogels that were left to 15 and 30 days (hydrate again) at a room‐temperature was characterized. Figure 4g shows the physical picture of *h*‐CA‐PAM‐Li^+^‐1.0 hydrogel placed for different times. Following a 15‐day period of storage at room temperature (24 (C), the length of *h*‐CA‐PAM‐Li^+^‐1.0 hydrogel was found to have decreased from 5 to 4 cm, due to the loss of water. However, after leaving for a certain days and rehydrated, the size of the hydrogel was restored to its original state. After 15 days of placement, the average SE_T_ of CA‐PAM‐Li^+^‐1.0 hydrogel decreased to 44.67 dB (Figure 4h). The decline in EMI shielding effectiveness can be attributed to the diminution of water content within the hydrogel. This reduction in water leads to the restriction of conductive ion movement within the hydrogel, thereby minimizing conductive and polarization loss of EMWs.^[^ ^15^ ^]^ However, the EMI shielding performance of *h*‐CA‐PAM‐Li^+^ hydrogel remains superior to that of CA‐PAM hydrogel (11.40 dB) and CA‐PAM‐Li^+^‐1.0 hydrogel (30.29 dB) (Figure 4i). This phenomenon can be attributed chiefly to the high hygroscopicity of LiCl. The dehydrated *h*‐CA‐PAM‐Li^+^ hydrogel has been shown to be capable of effectively absorbing water molecules from the surrounding environment^[^ ^9^ ^]^ thus ensuring the maintenance of the conductivity and polarization loss properties. Concurrently, the hydrogel left for 30 days and rehydrated, resulting in an SE_A_ of 43.62 dB and an SE_R_ of 4.76 dB. The prepared *h*‐CA‐PAM‐Li^+^‐1.0 hydrogels have been shown to possess both stable and efficient EMI shielding properties.

**Figure 5:**
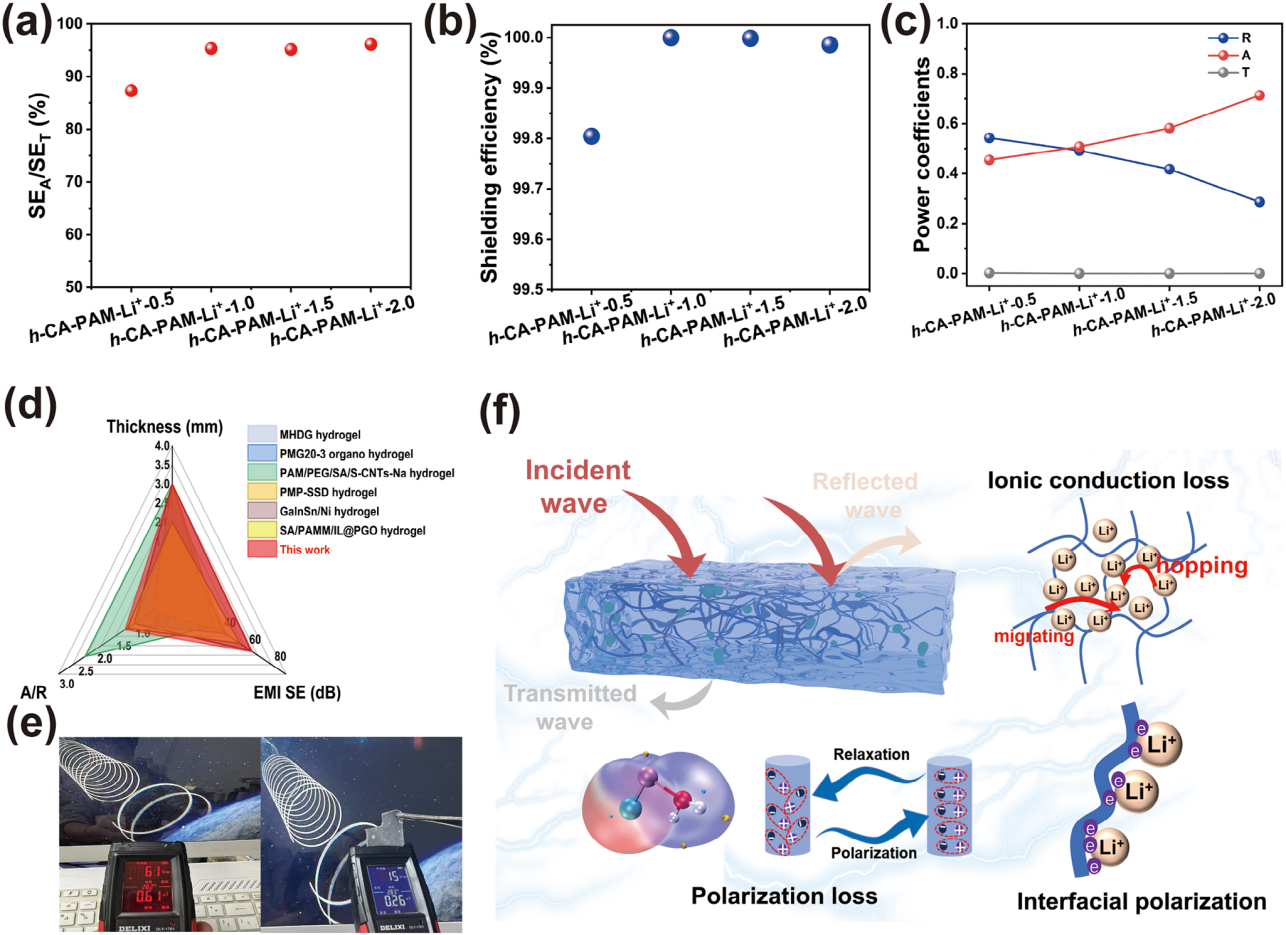
a) SE_A_/SE_T_. b) Shielding efficiency of *h*‐CA‐PAM‐Li^+^‐1.0 hydrogels. c) Power coefficients. d) Performance comparison chart of EMI shielding hydrogel. e) EMI shielding hydrogel practical applications. f) EMI shielding mechanism diagram of *h*‐CA‐PAM‐Li^+^ hydrogel.

To explore the EMI shielding mechanism of *h*‐CA‐PAM‐Li^+^ hydrogels, the contribution degree of SE_A_ to SE_T_ was analyzed (**Figure** **5**a). The SE_A_ of *h*‐CA‐PAM‐Li^+^ hydrogels was found to be greater than 85%, increasing to over 95% with the increase of LiCl, which was significantly higher than that of CA‐PAM‐Li^+^ hydrogels (81–93%). This indicates that the enhancement in conductivity is inadequate to augment the contribution of SE_R_ to SE_T_, thereby affirming that SE_A_ remains the predominant contributor. Consequently, the SE of *h*‐CA‐PAM‐Li^+^ hydrogels can reach more than 99.8%, which is larger than that of CA‐PAM‐Li^+^ hydrogels (98.0%) (Figure 5b). The SE of *h*‐CA‐PAM‐Li^+^‐1.0 hydrogel is as high as 99.9999%, which indicates that only 0.0001% of the EMWs escape, emphasizing its excellent EMI shielding performance. To show the principle of this shielding mechanism, A and R values of *h*‐CA‐PAM‐Li^+^ hydrogels were calculated (Figure 5c). Notably, *h*‐CA‐PAM‐Li^+^ hydrogels achieved A/R > 1 when the concentration of LiCl exceeded 1.0 m, which means they have an absorption‐dominated EMI shielding mechanism. Following the hydration treatment, the motility ability of Li^+^ is enhanced. The presence of an electromagnetic field results in the formation of a greater number of polarization effects, thereby enhancing the absorption loss of EMWs.^[^ ^13^ ^]^ Compared with recently reported hydrogels/organic hydrogels^[^ ^11^, ^12^, ^13^, ^31^, ^32^, ^33^ ^]^ (Figure 5d). The *h*‐CA‐PAM‐Li^+^‐1.0 hydrogel demonstrates superior EMI shielding performance at equivalent thicknesses, achieving significantly higher EMI SE values (63.75 dB). Crucially, it exhibits a remarkably higher absorption‐to‐reflection (A/R) ratio (1.25), realizing an absorption‐dominated EMI shielding mechanism, which aligns with the demand for greener EMI shielding solutions.

Additionally, the practical application effect of *h*‐CA‐PAM‐Li^+^ hydrogel in electronic product radiation was visually demonstrated. When there is no shielding between the radiation source and the detector, the electric and magnetic field radiation of the computer amounted to 61 V m^−1^ and 0.61 µT (Figure 5e). Interestingly, after shielding with *h*‐CA‐PAM‐Li^+^ hydrogel, the electromagnetic radiation from the laptop was reduced to within the safe range (40 V m^−1^ and 0.4 µT). Manifestly, *h*‐CA‐PAM‐Li^+^ hydrogel shows good commercial feasibility and stability.

In order to provide a more comprehensive illustration of the absorption‐based shielding mechanism realized by h‐CA‐PAM‐Li+ hydrogels, a schematic diagram of the mechanisms is provided in Figure 5f. The addition of lithium chloride helps to improve the electrical conductivity of the system and thus increases the reflection loss of the *h*‐CA‐PAM‐Li+ hydrogel.^[^ ^34^, ^35^, ^36^ ^]^ In addition, after the combined treatment, water molecules with a large dielectric constant cause an increase in the degree of polarization under the action of an electric magnetic field.^[^ ^29^ ^]^ This property promotes the enhancement of the ability of EMW energy conversion. Therefore, the introduction of water molecules significantly improves the polarization loss capability of hydrogels against EMWs.^[^ ^31^ ^]^ In addition, water molecules can increase the polarization interface through hydrogen bonding with polymers, thus enhancing the polarization loss capability of hydrogels against EMWs.^[^ ^10^ ^]^ Many heterogeneous interfaces are formed between PAM, CA, H_2_O, and Li^+^. Charge accumulates at these interfaces, and dipole polarization occurs within the hydrogel, leading to loss of interfacial polarization.^[^ ^37^, ^38^ ^]^ *h*‐CA‐PAM‐Li^+^ hydrogels achieve green absorption‐dominated EMI shielding behavior under the synergistic effect of multiple loss mechanisms.

**Figure 6:**
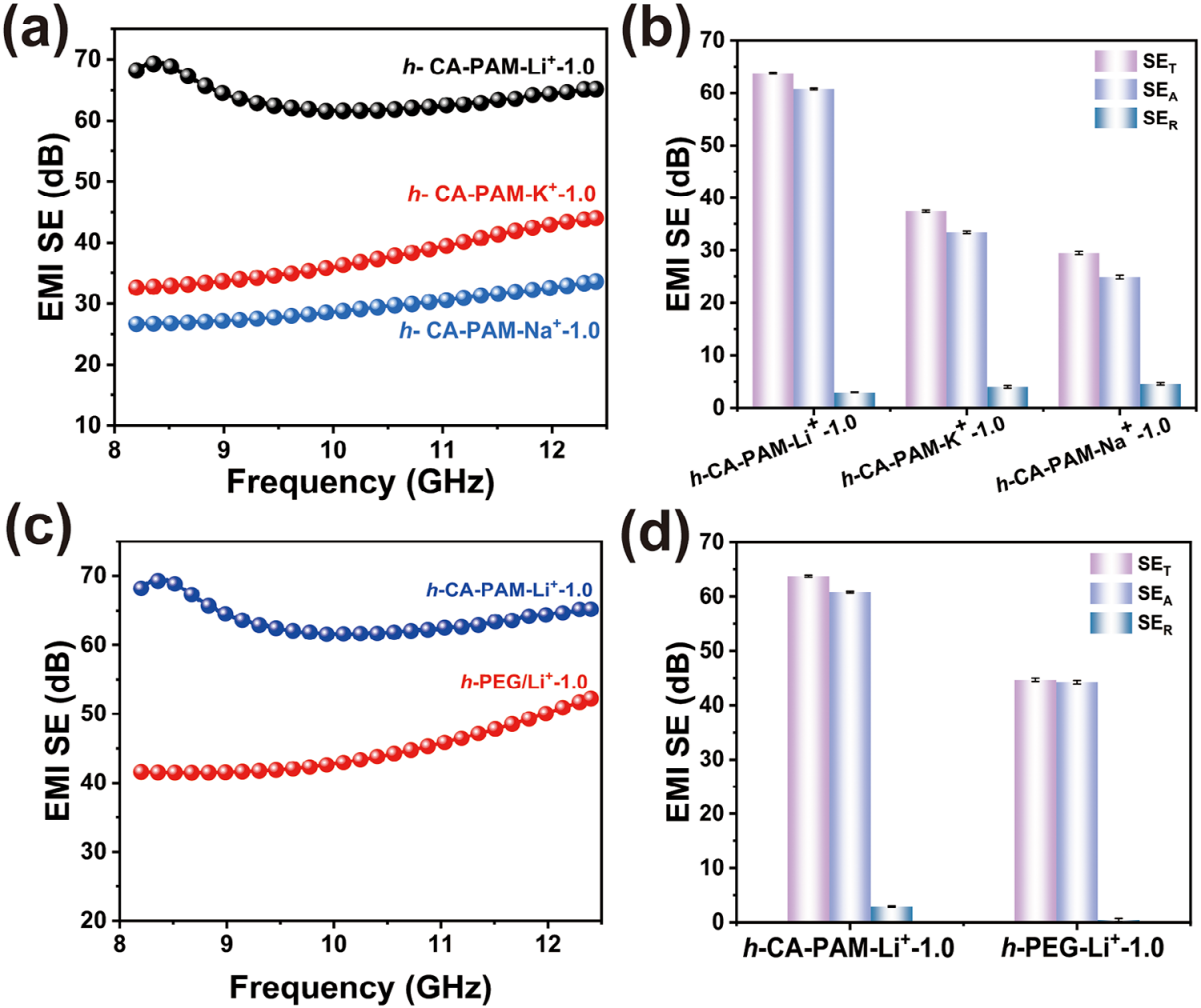
Universality of ion‐conducting hydrogel EMI shielding. a,b) EMI shielding performance of hydrogels with different conductive ionic. c,d) EMI shielding performance of different hydrogel matrices.

The enhanced EMI shielding performance of *h*‐CA‐PAM‐Li⁺ hydrogels mainly stems from their ionic conductivity, in which the synergistic effect of water‐mediated charge transport and ion‐cluster‐induced interfacial polarization plays a key role. However, the generalization of this approach requires further systematic studies. To achieve this objective, the *h*‐CA‐PAM‐K^+^ and *h*‐CA‐PAM‐Na^+^ hydrogels were prepared by loading KCl and NaCl into the hydrogels, respectively. The average SE_T_ of *h*‐CA‐PAM‐K^+^ and *h*‐CA‐PAM‐Na^+^ hydrogels was 37.49 and 29.48 dB (**Figure** **6**a,b). The results suggest that this mechanism may be extended to other ion‐doped hydrogel systems. Meanwhile, *h*‐PEG‐PAM‐Li^+^‐1.0 hydrogel was constructed by loading Li^+^ (1.0 m) in PEG and PAM hydrogels with SE_T_, SE_A_, and SE_R_ of 44.69, 44.26, and 0.42 dB, respectively (Figure 6c,d). The SE_A_ of *h*‐PEG‐PAM‐Li^+^‐1.0 hydrogel contributes up to 99.06% to the SE_T_. This indicates that the EMI shielding of conductive ionic hydrogel is primarily derived from the loss of internal EMWs. Furthermore, the A‐value of *h*‐PEG‐PAM‐Li^+^‐1.0 hydrogel is as high as 0.91 (Figure S8, Supporting Information), which exhibits an absorption‐based EMI shielding mechanism. Although the incorporation of K⁺ or Na⁺ into hydrogels imparts measurable EMI shielding performance, their SE remains substantially inferior to Li⁺‐Li‐incorporated counterparts. This performance hierarchy originates from the intrinsic differences in ionic conductivity and attenuation capability. *h*‐CA‐PAM‐Li^+^‐1.0 exhibits superior conductivity (≈40.69 S cm^−1^) compared to *h*‐CA‐PAM‐K⁺‐1.0 (≈25.64 S cm^−1^) and *h*‐CA‐PAM‐Na⁺‐1.0 (≈21.98 S cm^−1^) (Figure S9a, Supporting Information), primarily due to its smaller hydrated radius enabling faster ion mobility and reduced charge transport resistance. Consequently, the α(*h*‐CA‐PAM‐Li^+^‐1.0) > α(*h*‐CA‐PAM‐K⁺‐1.0) > α(*h*‐CA‐PAM‐Na⁺‐1.0) (Figure S9b, Supporting Information). This trend arises because higher conductivity directly amplifies ohmic loss, while the stronger polarization of *h*‐CA‐PAM‐Li^+^‐1.0 enhances interfacial charge accumulation at polymer networks, further boosting dielectric loss. These ion‐specific attributes collectively establish Li⁺ as the optimal cation for maximizing SE in conductive hydrogels, whereas the limited ion mobility and attenuated polarization of Na⁺/K⁺ restrict their energy dissipation efficiency.

Besides, metal salts with different ionic valences have also been used in the research. However, due to their rapid coordination with sodium alginate and the steric hindrance effect of the formed coordination compounds, the products gelate rapidly, which is not conducive to further processing, as shown in Figure S10 (Supporting Information). Moreover, their electrical conductivity and electromagnetic shielding performance are relatively poor. Therefore, no further in‐depth research has been conducted. The preceding analysis demonstrates that conductive ionic hydrogels represent an efficacious method of producing absorption‐oriented efficient EMI shielding materials.

### 2.4 Integration and Performance of EMW Protective Sensing Device

Good mechanical strength is essential for developing hydrogels as self‐powered sensors. Tensile tests were conducted on hydrogels with varying LiCl concentrations. As the LiCl concentration increased from 0.5 to 1.0 m, the strength of the h‐CA‐PAM‐Li^+^ hydrogel improved from 1.05 ± 0.08 to 1.69 ± 0.09 MPa (Figure S11, Supporting Information). This enhancement can be attributed to two primary factors. The increased ionic crosslinking density reinforces the sodium alginate (SA) primary network. Besides, a stronger Hofmeister effect promotes greater molecular entanglement within the SA network and between the SA and polyacrylamide (PAM) secondary networks. However, excessive LiCl addition accelerates the gelation of SA. Rapid gelation may lead to localized crosslinking before homogeneous mixing, resulting in an uneven network structure. Under tensile stress, such structural inhomogeneity can induce stress concentration zones, ultimately degrading mechanical performance.

**Figure 7:**
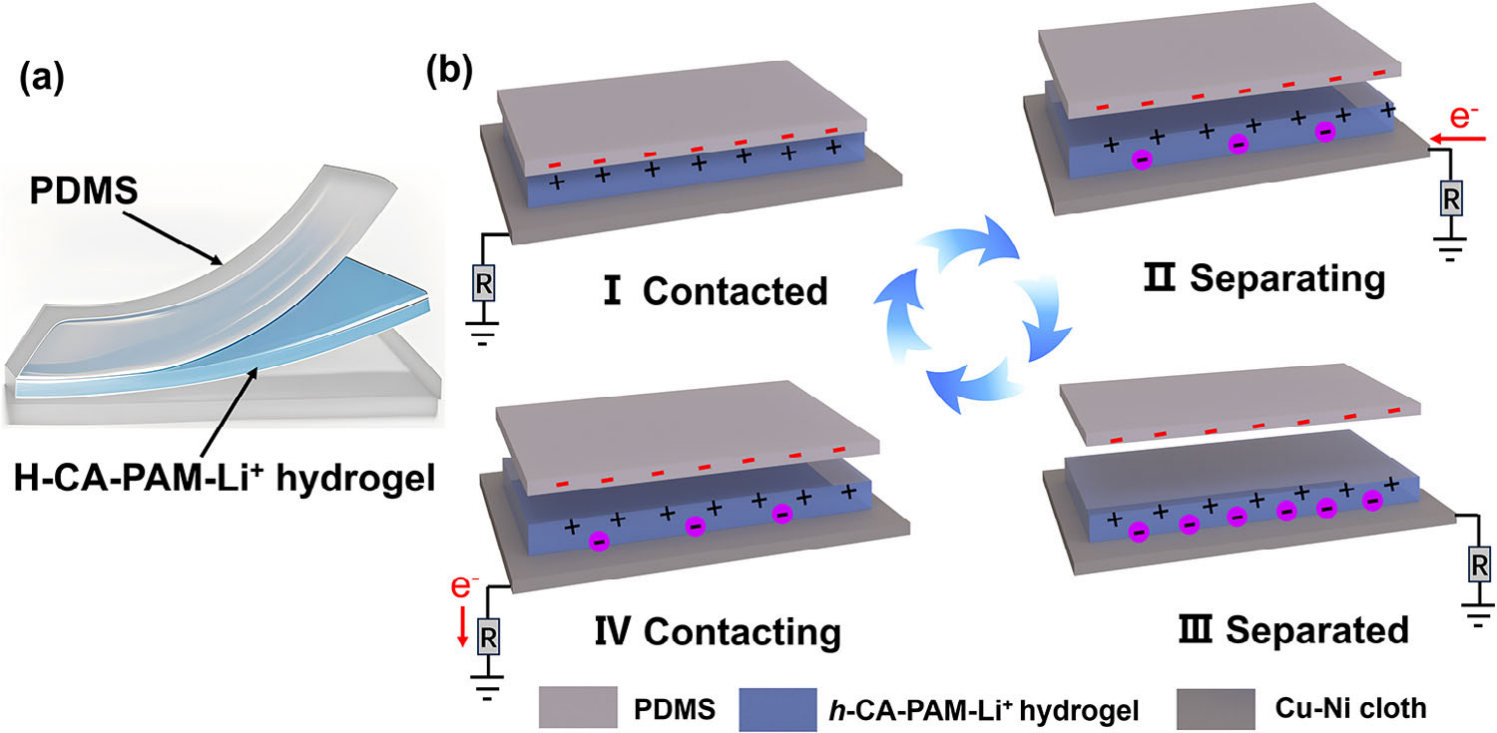
Principle of operation of hydrogel self‐powered sensors. a) A schematic structure of the hydrogel self‐powered sensors, b) A schematic working principle of the single‐electrode mode self‐powered sensors for energy harvesting.

To realize an integrated protective monitoring device suitable for cardiovascular implantable electronic equipment, a single‐electrode self‐powered sensor was constructed using *h*‐CA‐PAM‐Li⁺ hydrogel encapsulated with a thin PDMS layer as the electrode based on the triboelectric effect (**Figure** **7**a). The working mechanism relies on the coupling of triboelectrification and electrostatic induction^[^ ^39^, ^40^ ^]^ (Figure 7b). At the initial state (Figure 7bI), when an external force induces full contact between PDMS and *h*‐CA‐PAM‐Li⁺ hydrogel, triboelectric charges are generated at their interface. Due to the higher electron affinity of PDMS in the triboelectric series, it retains negative surface charges, while the *h*‐CA‐PAM‐Li⁺ hydrogel acquires positive charges. As PDMS separates from the hydrogel, the electrostatic equilibrium is disrupted. The positive charges on the hydrogel induce a redistribution of electrons in the conductive Cu‐Ni cloth (electrode), attracting free anions in the hydrogel toward its upper surface. This generates a transient current with electrons flowing from the Cu‐Ni cloth to the hydrogel through the external circuit (Figure 7bII). At maximum separation, the electric field between PDMS and the hydrogel weakens due to increased distance. The induced charges on the Cu‐Ni cloth reach electrostatic equilibrium, halting further electron transfer in the circuit (Figure 7bIII). When the Cu‐Ni cloth approaches PDMS again, the reduced distance strengthens the electric field. The anions in the hydrogel are repelled downward, while electrons in the external circuit flow back toward the ground to balance the potential difference (Figure 7bIV). This cyclic process converts mechanical energy into electrical signals, enabling self‐powered sensing.

**Figure 8:**
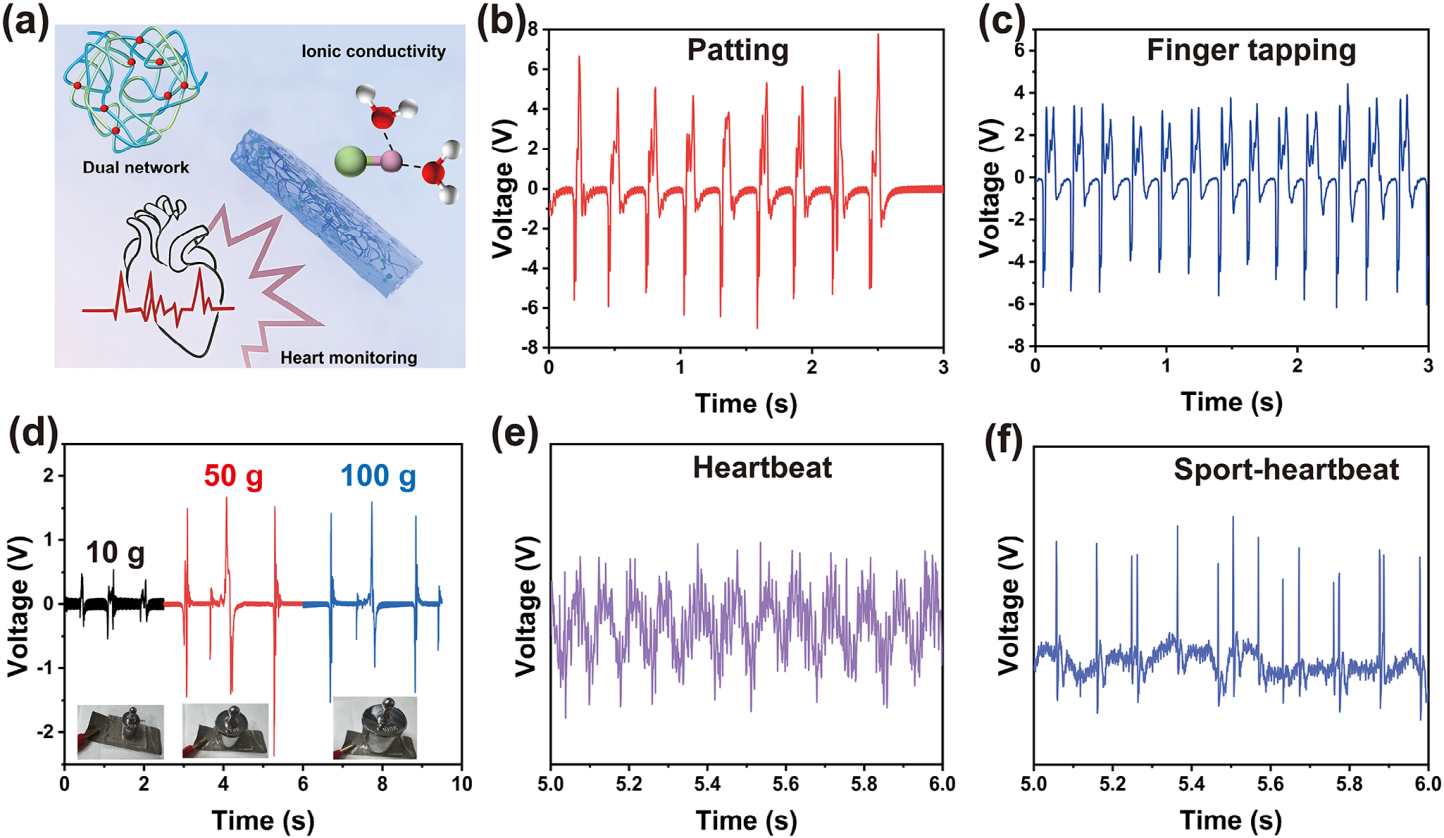
a) The advantages and flexible sensing applications of *h*‐CA‐PAM‐Li⁺ hydrogel. b) Hand patting. c) Finger tapping. d) Sensing tests with different weights. e) and f) Heartbeat monitoring during calm and exercise.

The prepared EMWs protective sensing electrode, after being integrated with the wireless signal transmission module, can be used for monitoring various physiological signals of the human body. (**Figure** **8**a). The dynamic response characteristics of the sensor were systematically evaluated through multi‐scenario mechanical tests, as illustrated in Figure 8b and d. In the palm‐tapping experiment (Figure 8b), the contact‐separation process between the PDMS triboelectric layer and the hydrogel electrode efficiently converted mechanical impact into voltage pulses (Video S1, Supporting Information). The signal amplitude exhibited a direct correlation with the applied force, thereby confirming the high sensitivity of the sensor and rapid response capability. Further finger‐tapping tests (Figure 8c) revealed that the sensor could precisely detect variations in the friction interface's contact area induced by subtle deformations, generating stable electrical signals corresponding to tapping frequency and intensity (Video S2, Supporting Information). These results underscore their potential for haptic feedback systems and low‐frequency motion recognition. To assess static pressure sensing performance, experiments with varying weights (Figure 8d) demonstrated that the sensors’ output signals exhibited significant changes proportional to the applied loads (10, 50, and 100 g), highlighting their reliable static pressure sensing performance suitable for wearable pressure distribution monitoring.

Beyond mechanical sensing, the self‐powered sensor fabricated from *h*‐CA‐PAM‐Li⁺ hydrogel demonstrated robust capability in physiological signal detection for health monitoring applications (Figure S12, Supporting Information). Heartbeat monitoring experiments (Figure 8e,f) validated its clinical relevance. In the calm state (Figure 8e), the sensor attached to the chest captured periodic heartbeat vibrations with sinusoidal waveforms and a high signal‐to‐noise ratio (Video S3, Supporting Information). During motion (Figure 8f), despite baseline fluctuations caused by body movement, the sensor successfully extracted accelerated heartbeat signals through adaptive filtering algorithms while maintaining stable peak resolution (Video S4, Supporting Information). This performance confirmed its anti‐interference capability and operational robustness in dynamic environments.

## 3 Conclusion

This study develops an ionic double‐network hydrogel with efficient electromagnetic absorption and self‐powered sensing capabilities through material design‐mechanism regulation‐device integration, offering a breakthrough solution for electromagnetic compatibility issues in implantable electronic devices. The double‐network skeleton, formed by SA/Ca^2^⁺ physical crosslinking and AM chemical polymerization, synergizes with Li⁺ coordination to construct ordered ionic transport channels, overcoming the limitation of single‐loss mechanisms in traditional hydrogels and achieving synchronous optimization of electrical conductivity (up to 51.48 S cm^−1^) and dielectric loss. By regulating ionic concentration gradients and hydration treatment, an absorption‐dominated shielding mechanism is established, avoiding reflection pollution and biocompatibility risks of metal‐based materials. With a thickness of 3 mm, *h*‐CA‐PAM‐Li⁺‐1.0 achieves an EMI shielding effectiveness (SE_T_) of 63.75 dB, outperforming most reported conductive hydrogel systems. Leveraging the hydrogel's ionic conductivity, self‐powered sensing electrodes are fabricated to enable seamless integration of electromagnetic protection and physiological signal (e.g., heartbeat) monitoring, demonstrating real‐time and precise dynamic response in wearable medical devices. The ionic doping strategy is extendable to various conductive ions (K⁺, Na⁺) and matrix systems (PEG‐PAM), verifying its universal mechanism. Free of noble metal fillers and relying on low‐cost hydration processing, this approach aligns with the trend of green materials development, providing critical insights for designing advanced functional materials.

## 4 Experimental Section

### Materials

Sodium alginate (SA) and lithium chloride anhydrous (LiCl) were purchased from Aladdin. Acrylamide (AM), ammonium persulphate (APS), calcium sulphate (CaSO_4_·H_2_O), sodium chloride, and potassium chloride were purchased from Macklin. N,N″‐methylenebisacrylamide (MBAA) and N,N,N″,N′‐tetramethylethylenediamine (TEMED) were purchased from Sigma‐Aldrich.

### Preparation of Hydrogels

Dissolve 0.25 g of sodium alginate in 12.5 mL of deionised water by heating and stirring. Then, acrylamide (2.00 g) was added into the dissolved solution and shaken until sodium alginate and acrylamide were fully mixed. After degassing for 10 min, add different concentrations of LiCl. Then, 60 µL 0.10 g mL^−1^ APS aqueous solution, 96 µL 0.025 g mL^−1^ MBAA aqueous solution and 10 µL TEMED were added. In this system, APS is the photo‐thermal‐initiator, MBAA is the crosslinking agent, and TEMED is the crosslinking accelerator. Subsequently, 4 mL CaSO_4_·2H_2_O (0.0221 g) aqueous slurry was added as an ionic crosslinker for alginate. The resulting solution was poured into a plastic mould and cured with UV light for 10 min. Subsequently, the cured mixture was left in the mould for several hours to stabilize. Conducting hydrogels prepared at 0.5, 1.0, 1.5, and 2.0 m LiCl concentrations were named CA‐PAM‐Li^+^‐0.5, CA‐PAM‐Li^+^‐1.0, CA‐PAM‐Li^+^‐1.5, and CA‐PAM‐Li^+^‐2.0. Hydration of hydrogels using salt solutions and the samples were named *h*‐ CA‐PAM‐Li^+^‐0.5, *h*‐ CA‐PAM‐Li^+^‐1.0, *h*‐ CA‐PAM‐Li^+^‐1.5, and *h*‐ CA‐PAM‐Li^+^‐2.0.

### Characterization

For characterization of the micro‐ and nanostructure of these hydrogels, which were freeze‐dried using a freeze‐dryer. The freeze‐dried gels were cut along the aligned direction to expose their interior. The morphological structure was observed using scanning electron microscopy (SEM, EVO18, Zeiss, Germany). The chemical structures were characterized by Attenuated Total Reflectance Fourier transform infrared (FTIR). The hydrogel samples were molded into dumbbell shapes (25 × 4 × 2 mm) using PTFE molds and then subjected to tensile testing on a universal mechanical testing machine (MT 5504, Shenzhen SASTEST Cooperation, China) at a pulling rate of 10 mm min^−1^. This process yielded tensile strength for the *h*‐CA‐PAM ‐Li^+^ hydrogels with different LiCl content, where each sample underwent a minimum of three repeated tests. The ionic conductivities (σ) of the gels in the relaxed state were determined by the AC impedance spectroscopy with an electrochemical workstation system (CHI660E) equipped with a typical three‐electrode system. The ionic conductivity was then calculated according to Equation (3):

(3) $$\sigma =\frac{L}{A\times R}$$

where σ is the ionic conductivity, L is the separation between the two electrodes, R is the resistance and A is the area. PDMS‐encapsulated hydrogel samples of 30 × 20 × 3 mm were connected to an eight‐channel TENG/PENG integrated wireless collector, and the physiological signals were visualized and monitored in real‐time by Labviwe software.

The scattering parameters (S_11_ and S_21_), complex permittivity (ε′ and ε′′), and permeability (µ′ and µ′′) were measured by a vector network analyzer (VNA, N5244A, Agilent, USA) in the frequency range of 8.2–12.4 GHz (X‐band) using the waveguide method, the tested MBC composite materials were processed into a regular rectangle block with a 3D size of 22.86 mm × 10.16 mm × 3 mm. The corresponding reflection coefficient (R), penetration coefficient (T), absorption coefficient (A), reflection loss (SE_R_), absorption loss (SE_A_), and total EMI (SE_T_) were calculated according to S_11_, S_21_, ε′, ε′′, µ′, and µ′′ by the following Equations (4)–(9):

(4) $$R={\left|S_{11}\right|}^{2}$$

(5) $$T={\left|S_{21}\right|}^{2}$$

(6) A=1−R−T

(7) $$SE_{R}\;\left(dB\right)=10\log\left(\frac{1}{1-|S_{11}{|}^{2}}\right)$$

(8) $$SE_{A}\;\left(dB\right)=10\log\left(\frac{1-|S_{11}{|}^{2}}{|S_{21}{|}^{2}}\right)$$

(9) $$SE_{T}=SE_{R}+SE_{A}$$

### Density Function Theory (DFT) Calculations

DFT calculations details are placed in the supporting information.

## Conflict of Interest

The authors declare no conflict of interest.

## Author Contributions

C.W. performed wrote the original draft, methodology, formal analysis, and data curation. Y.D. and T.W. performed methodology, investigation, and data curation. Z.L. performed the methodology, investigation. C.H. performed supervision, project administration. Z.W. and Y.Z. performed an investigation. X.L. performed resources, wrote, reviewed, and edited. W.Z. performed the Investigation and formal analysis. J.X. performed Supervision, Project administration, wrote, reviewed, and edited, and visualization.

## Supporting information



Supporting Information

Supplemental Video 1

Supplemental Video 2

Supplemental Video 3

Supplemental Video 4

## Data Availability

The data that support the findings of this study are available in the supplementary material of this article.
