# Supplementary material for: Ionic Double‐Network Hydrogels for Integrated Electromagnetic Shielding and Self‐Powered Sensing in Wearable Electronics
Source: Adv Sci (Weinh). 2025 Jun 30;12(37):e09115. doi: 10.1002/advs.202509115 (PMC12499480; doi:10.1002/advs.202509115)
Supplement: Supplementary file 1 — Supporting Information [file ADVS-12-e09115-s004.docx]

**Ionic Double-Network Hydrogels for Integrated Electromagnetic Shielding and Self-Powered Sensing in Wearable Electronics**

*Chenchen Wang, Yao Ding, Tianzhao Wu, Zihua Li, Chuanshuang Hu*, Zhuoqun Wang, Yonghui Zhou, Xiuyi Lin*, Weiwei Zhang, Jiangtao Xu**

C. Wang, Y. Ding, C. Hu, Z. Wang, Y. Zhou, X. Lin, W. Zhang, J. Xu

Key Laboratory of Advanced Materials for Facility Agriculture，Ministry of Agriculture and Rural Affairs, College of Materials and Energy, South China Agricultural University, No. 483 Wushan Road, Guangzhou 510642, China

E-mail：cshu@scau.edu.cn (C. Hu), lxysandy@scau.edu.cn (X. Lin), jiangtao.xu@scau.edu.cn (J.Xu)

T. Wu

State Key Laboratory of Advanced Forming Technology and Equipment, China Academy of Machinery Science and Technology, No. 2 Shouti South Road, Beijing 100000, China

Z. Li

Academy of Interdisciplinary Studies on Intelligent Molecules, Tianjin Key Laboratory of Structure and Performance for Functional Molecules, College of Chemistry, Tianjin Normal University, Tianjin 300387, China


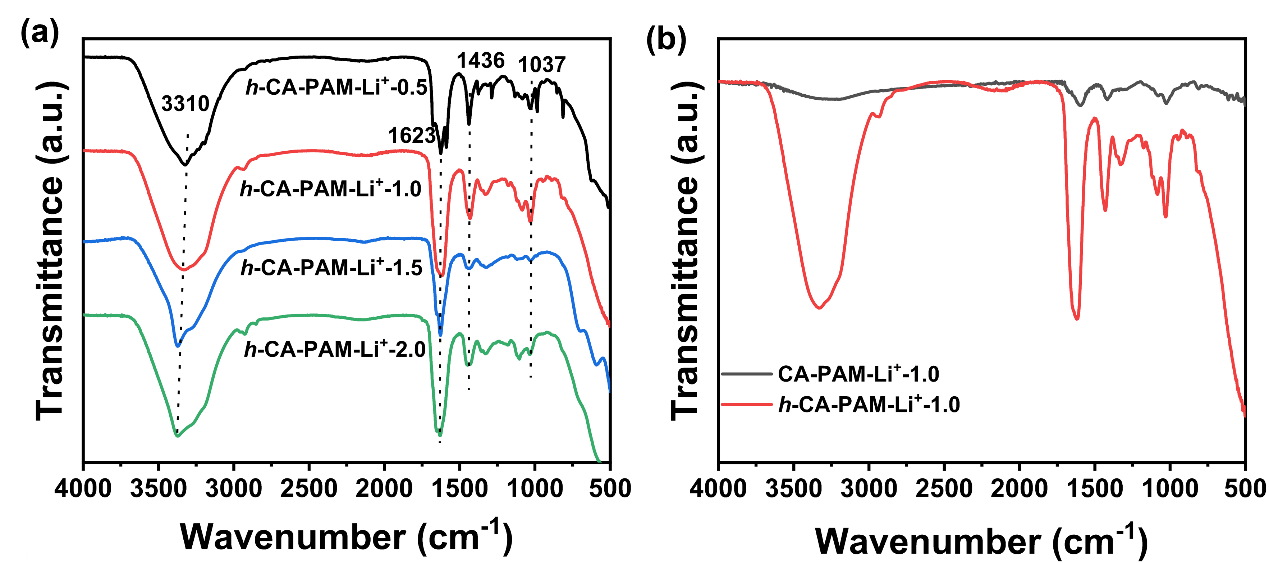


**Figure S1** The FTIR of (a) *h*-CA-PAM-Li^+^ hydrogels with different LiCl contents with different LiCl contents and (b) CA-PAM-Li^+^ -1.0 hydrogels before and after hydration.

**
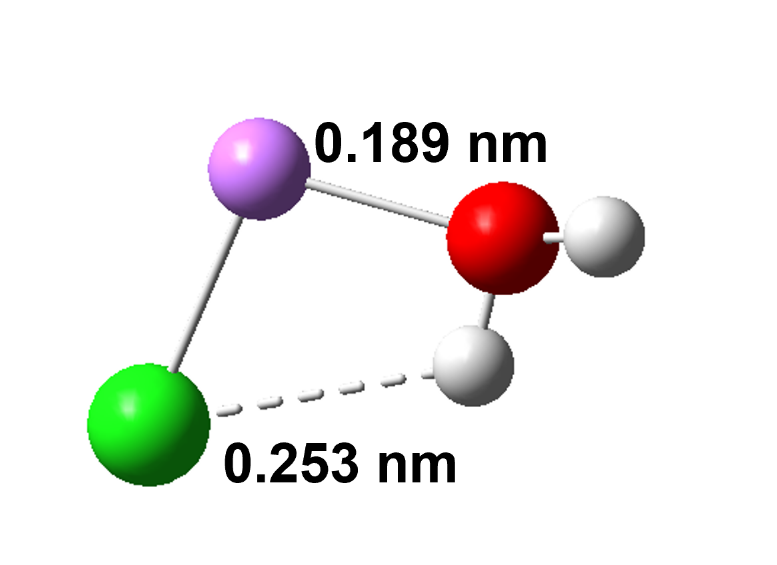
**

**Figure S2** Average bond lengths of the ground-state structures (LiCl-H_2_O).

**
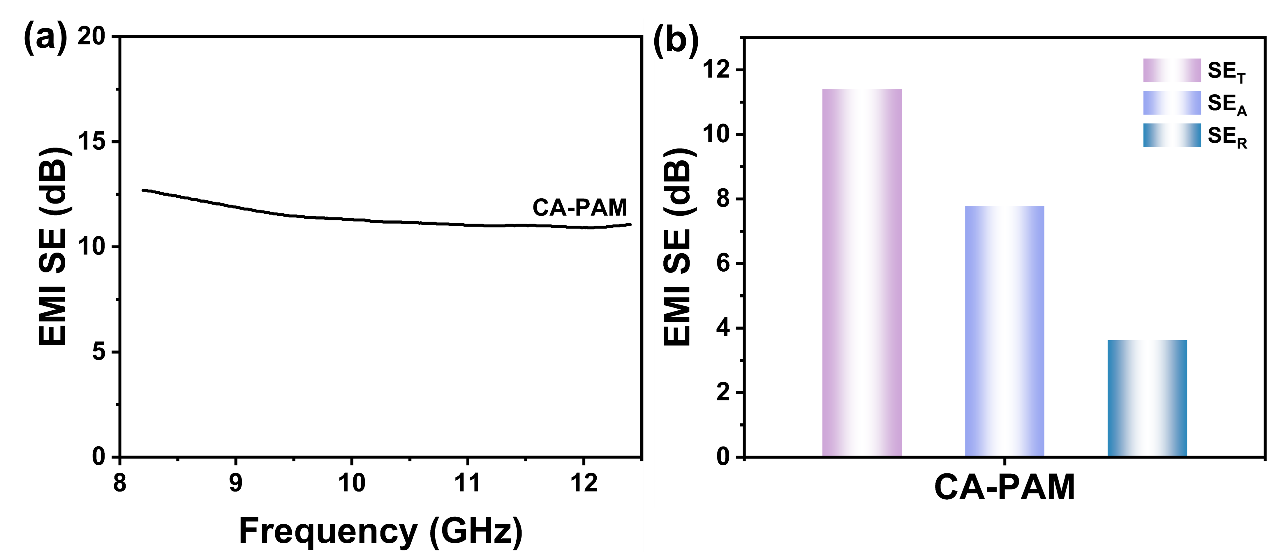
**

**Figure S3** The EMI shielding properties of CA-PAM hydrogel. (a) SE. (b) SE_T_, SE_A_, and SE_R._

**
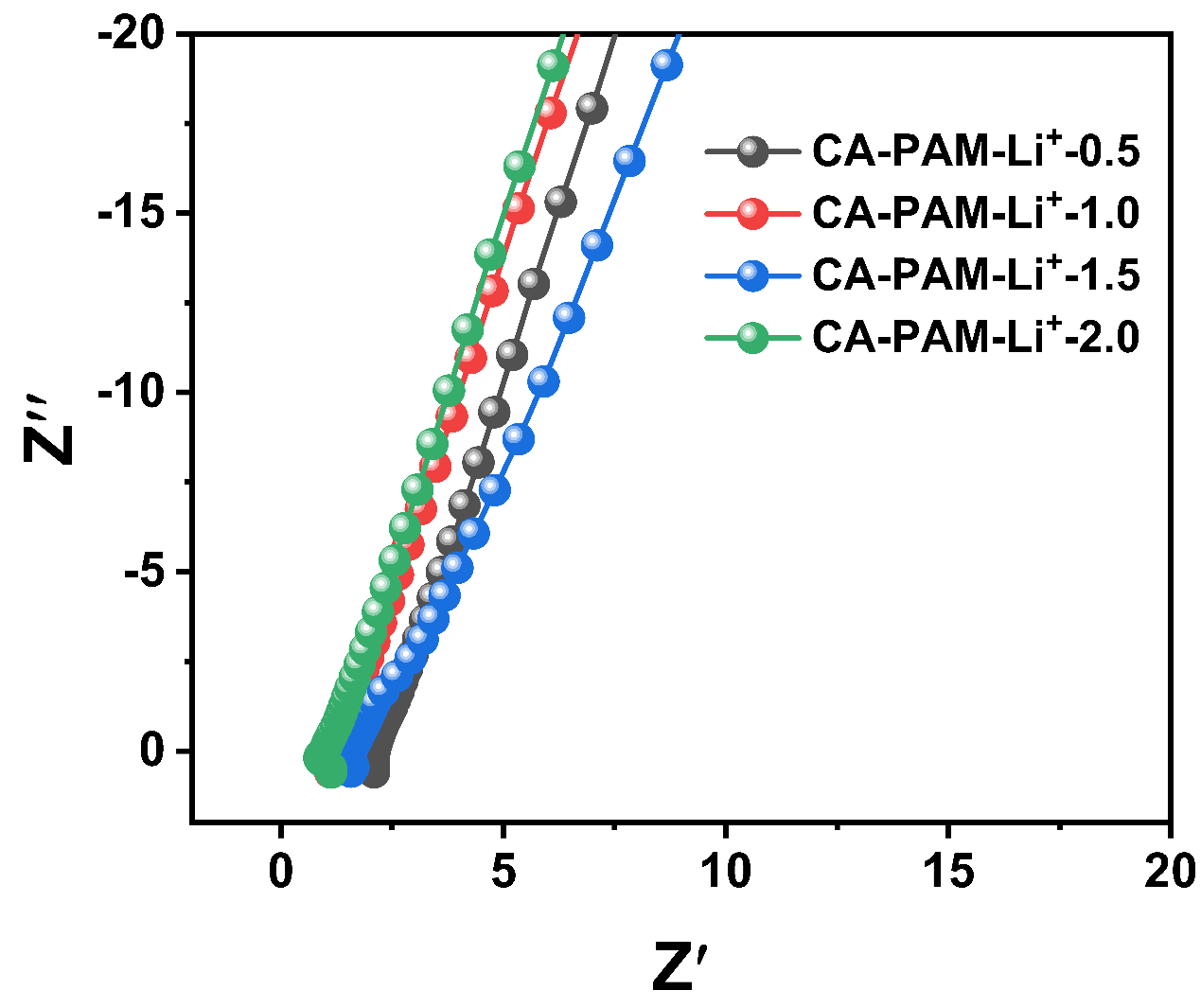
**

**Figure S4** Nyquist diagrams of CA-PAM-Li^+^ hydrogels with different LiCl contents with different LiCl contents.


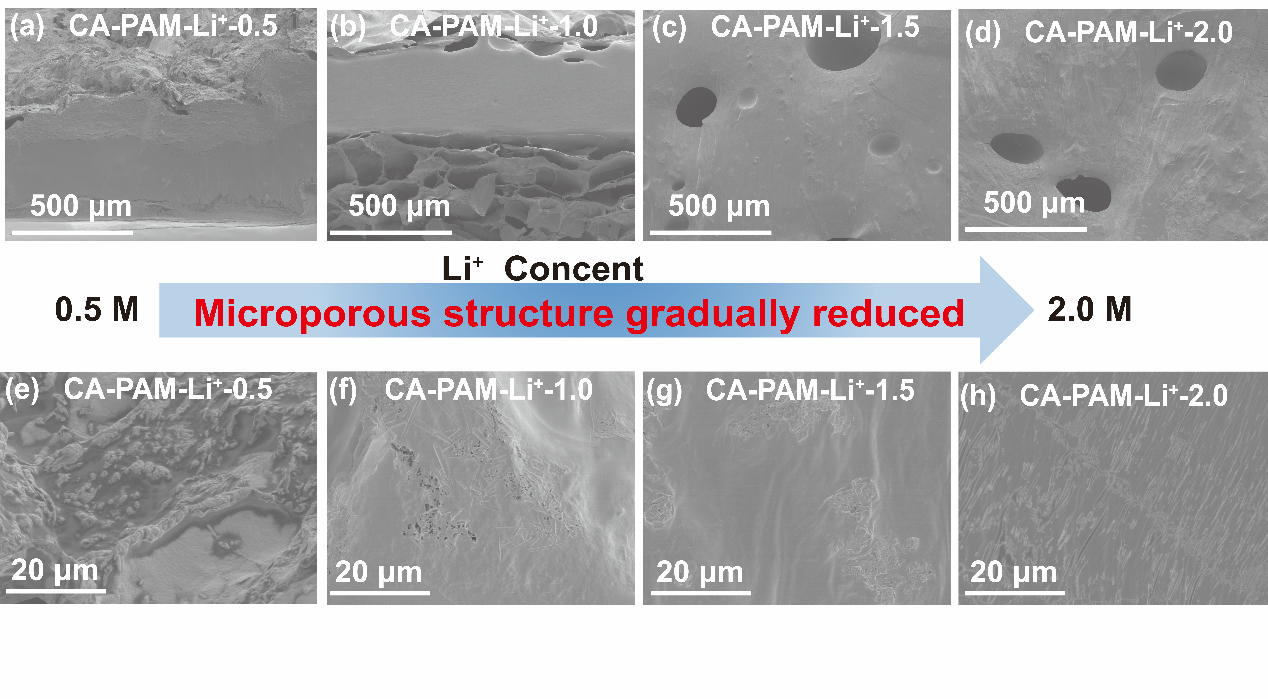


**Figure S5** The microstructure changes of CA-PAM-Li^+^ hydrogels. (a,e) CA-PAM-Li^+^-0.5, (b,f) CA-PAM-Li^+^-1.0, (c,g) CA-PAM-Li^+^-1.5, (d,h) CA-PAM-Li^+^-2.0.


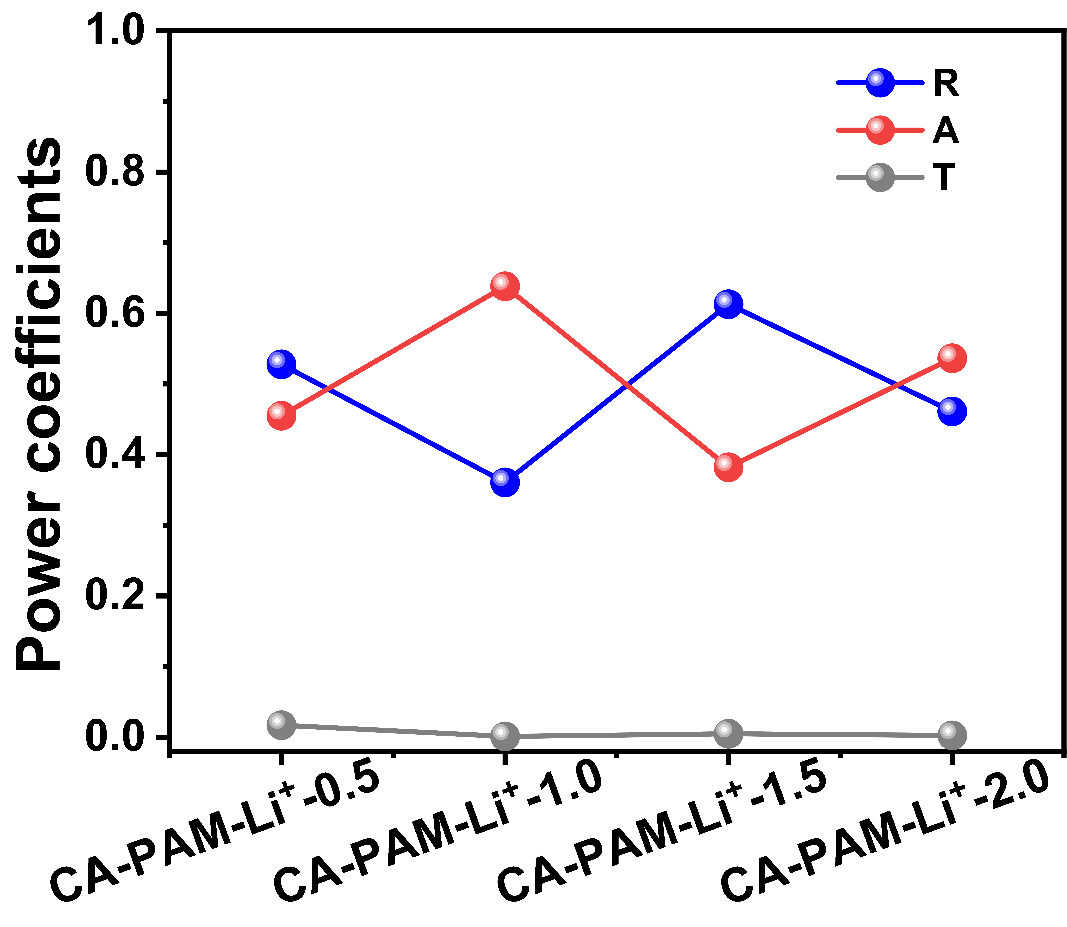


**Figure S6** A, R, and T coefficients of CA-PAM-Li^+^ hydrogels with different LiCl contents.


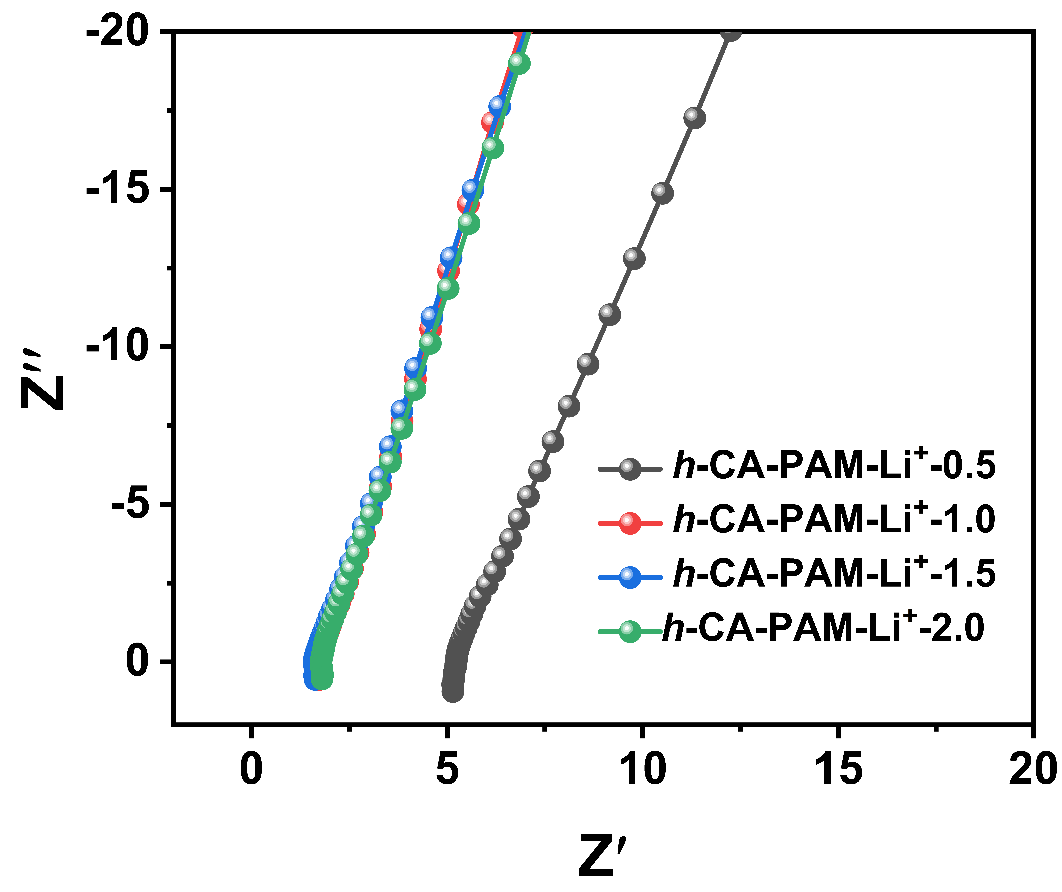


**Figure S7** Nyquist diagrams of *h*-CA-PAM-Li^+^ hydrogels with different LiCl contents with different LiCl contents.


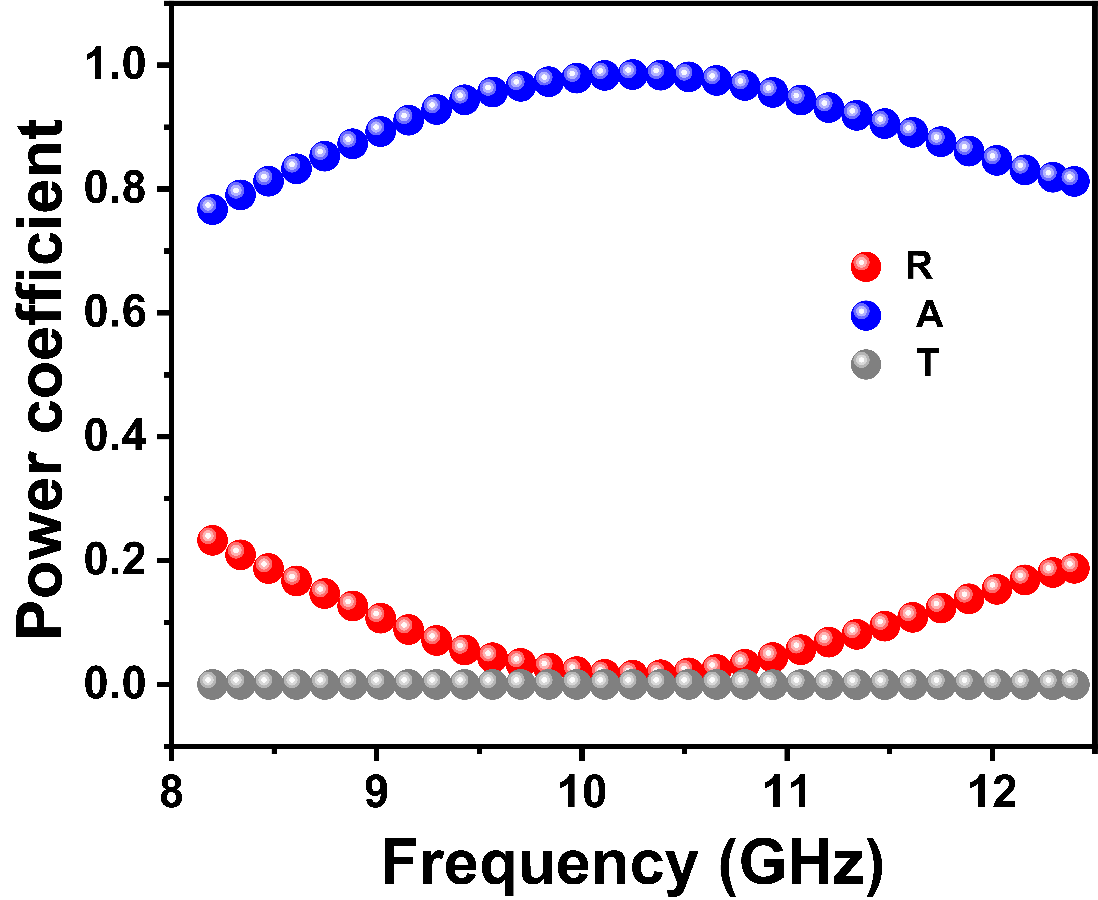


**Figure S8** A, R, and T coefficients of *h*-PEG/Li^+^-1.0 hydrogel.

**
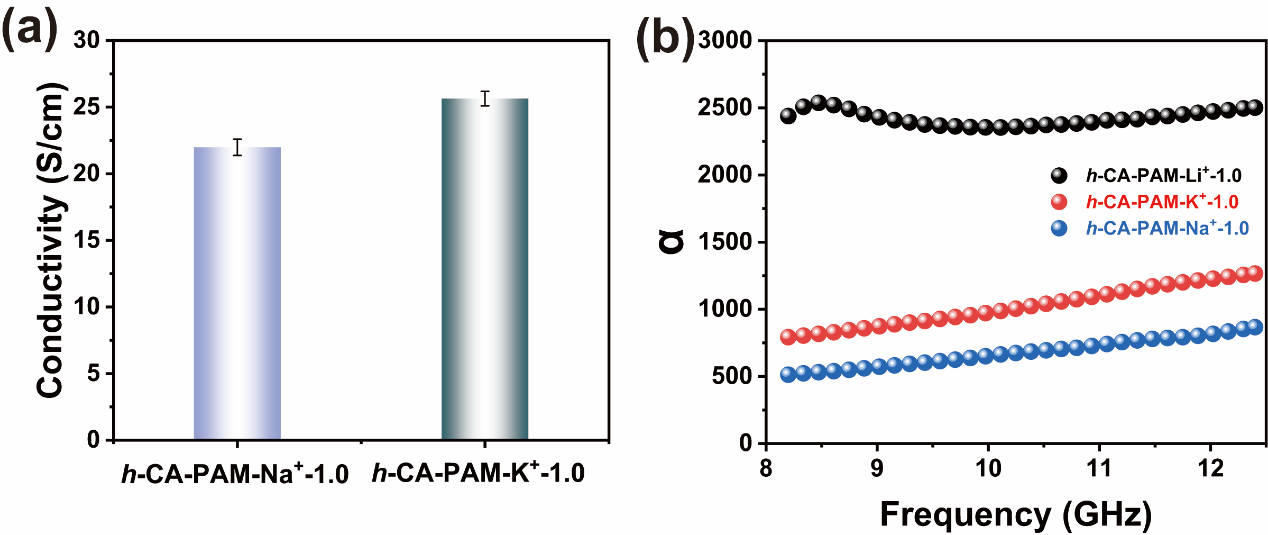
**

**Figure S9** (a) Conductivity and (b) α of *h*-CA-PAM-K^+^-1.0 and h-CA-PAM-Na^+^-1.0 hydrogels.

**
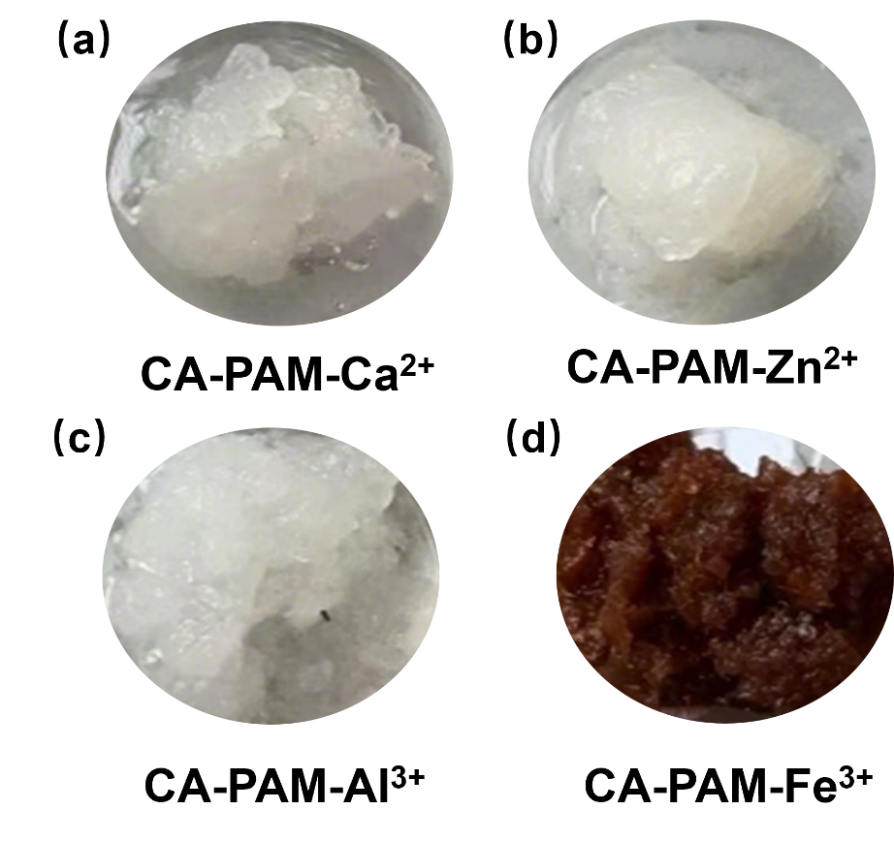
**

**Figure S10** Preparation of conductive hydrogels from metal salts with different ionic valences. (a) CA-PAM-Ca^2+^，(b) CA-PAM-Zn^2+^，(c) CA-PAM-Al^3+^，(d)CA-PAM-Fe^3+^.

Sodium alginate is crosslinked through the “egg carton” structure of divalent cations (e.g., Ca²⁺, Zn²⁺), but when the concentration of divalent cations is too high, it leads to rapid crosslinking, which is detrimental to its molding (Figure S10a and b). In contrast, excessive Al³⁺ charge density may trigger excessive cross-linking of the sodium alginate chains, leading to localized precipitation rather than uniform gels (Figure S10c). In addition, Fe³⁺ is susceptible to hydrolysis under neutral or alkaline conditions to form hydroxide precipitates (Fe(OH)₃), which lose their cross-linking ability (Figure S10d).


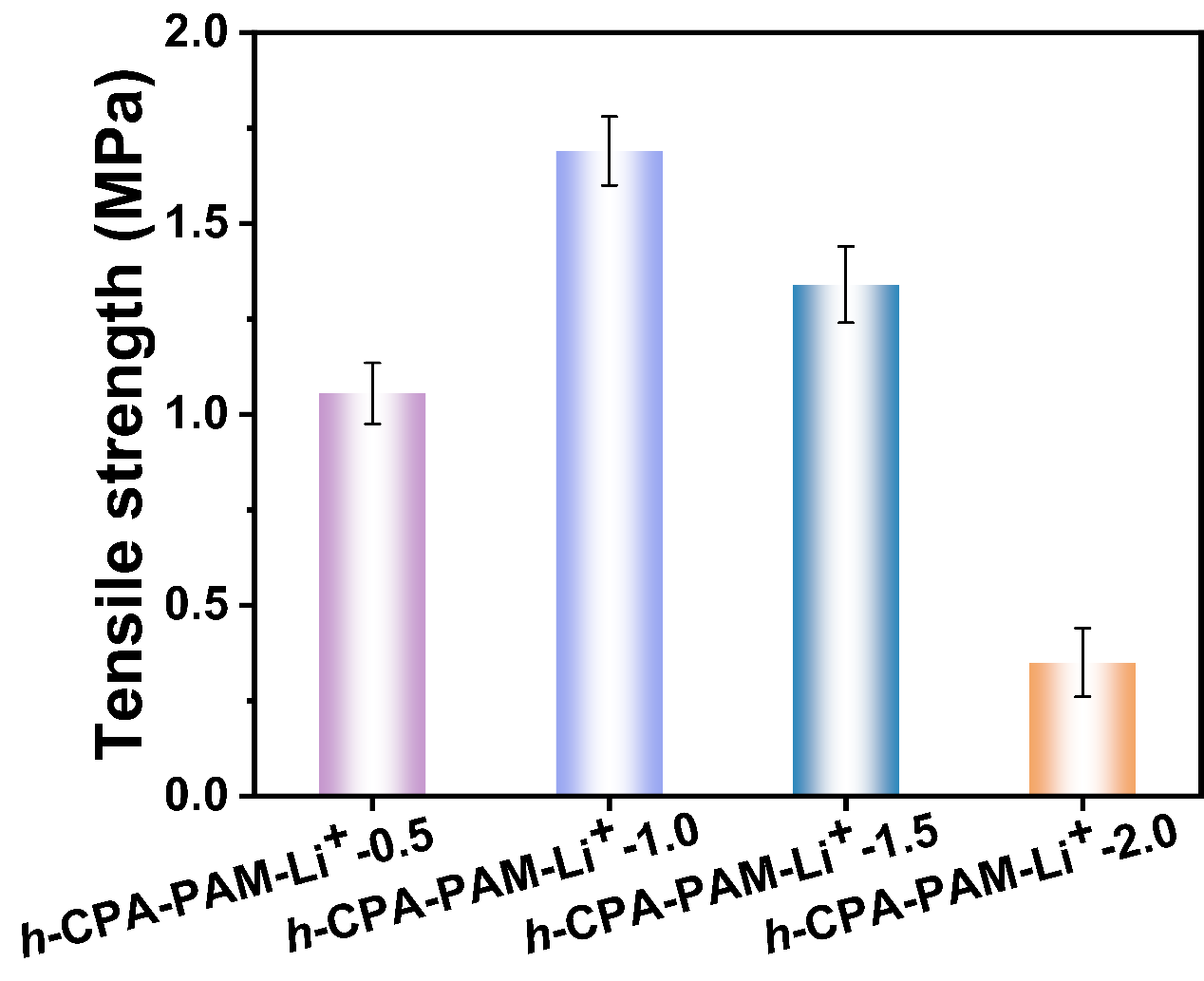


**Figure S11** Tensile properties of *h*-CA-PAM-Li^+^ hydrogel.


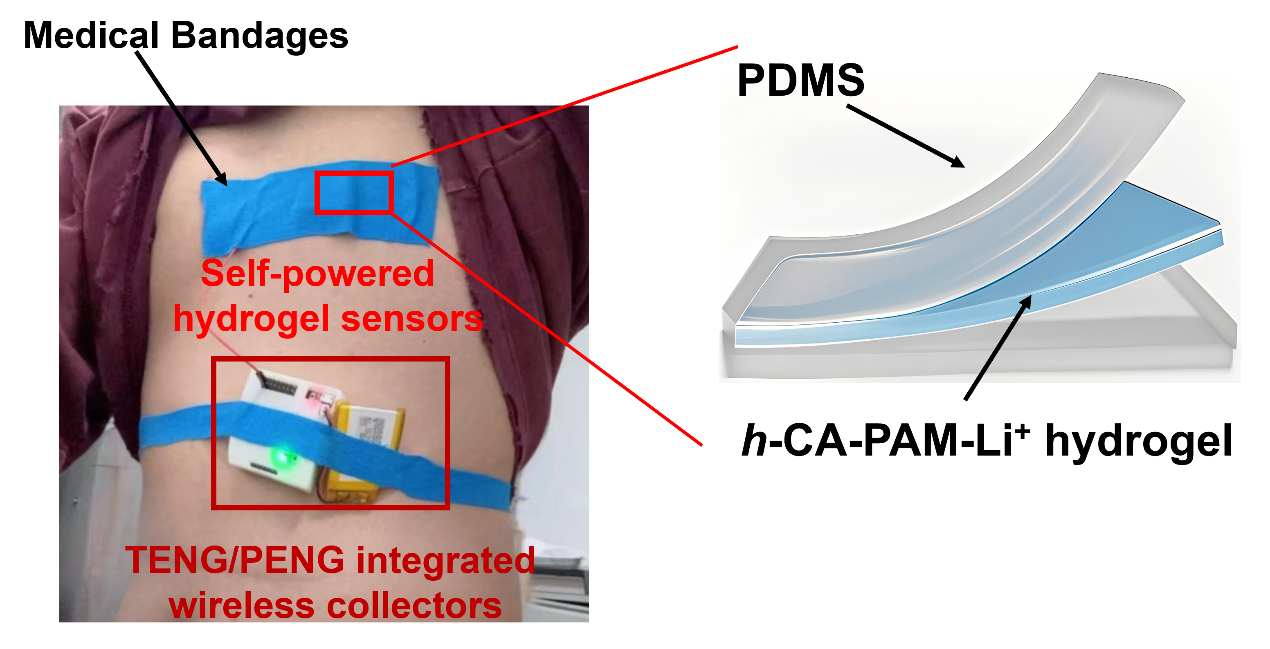


**Figure S12** Diagram of the actual device of a self-powered hydrogel sensor for physiological signal detection.

**Density function theory (DFT) calculations**

The molecular structures of H_2_O, LiCl, LiCl-H_2_O, KCl-H_2_O, and NaCl-H_2_O were prepared with GaussView 6.0 and submitted to the Gaussian 09 program ^[S1, S2]^ for structure optimization and frequency calculations based on the B3LYP-D3/6-311 + G (d, p) calculated level. In order to obtain reliable structures for H_2_O, LiCl, and LiCl-H_2_O, a multiframe file containing all the initial structures was generated using the genmer program ^[S3]^ and the one with the lowest energy was selected for frequency calculations. The wavefunction calculations were done in the Multiwfn 3.8 program ^[S4]^ and the visualization of the ESP was performed by the VMD 1.9.3 software.

**References**

1. T. Wu, T. Sun, Y. Ren, R. Zhang, Molecular Mechanism for the Absorption of Ketone Volatile Organic Compounds by Ionic Liquids, *Computational and Theoretical Chemistry* **2024**, *1234*, 114545.
2. F. Yang, T. Wu, H. Xin, M. Lv, Y. Wang, H. Fang, H. Chen, L. San, Q. Zhang, Z. Zhang, Separation of Methyl Ethyl Ketone-Methanol Azeotrope by Ionic Liquids: Thermodynamic and Molecular Mechanism, *Chemical Engineering Science* **2023**, *282*, 119314.
3. Z. Chen, Y. Zhang, M. Zhou, K. Yin, Y. Zhou, P. Cui, Z. Zhu, L. Zhong, Y. Wang, Mechanism Analysis and Process Optimization of Acetone–Methanol Azeotrope Separation Using 1-Ethyl-3-Methylimidazolium Acetate Based Mixed Extractants, Journal of Cleaner Production **2022**, *379*, 134687.
4. T. Lu, F. Chen, Multiwfn: A multifunctional wavefunction analyzer, Journal of Computational Chemistry **2012**, *33*, 580-592.
